# *Cucurbita pepo* var. *styriaca* Seeds: Deep Insights into Polar Lipid Profile

**DOI:** 10.3390/foods15122215

**Published:** 2026-06-19

**Authors:** Annunziata Paolillo, Assunta Napolitano, Francesco Sottile, Milena Masullo, Sonia Piacente

**Affiliations:** 1 Dipartimento di Farmacia, Università degli Studi di Salerno, Via Giovanni Paolo II n. 132, 84084 Fisciano, SA, Italy; anpaolillo@unisa.it (A.P.); mmasullo@unisa.it (M.M.); 2 PhD Program in Drug Discovery and Development, Università degli Studi di Salerno, Via Giovanni Paolo II n. 132, 84084 Fisciano, SA, Italy; 3 Dipartimento di Architettura, Università degli Studi di Palermo, Piazza Marina 61, 90133 Palermo, PA, Italy; francesco.sottile@unipa.it; 4 National Biodiversity Future Center (NBFC), 90133 Palermo, PA, Italy

**Keywords:** *Cucurbita pepo* var. *styriaca* seeds, polar lipids, sphingolipids, triterpenoids, LC-HRMS/MS analysis

## Abstract

The edible seeds of pumpkin plants (genus *Cucurbita*) are becoming increasingly appreciated as functional foods for their nutritional benefits, medicinal properties, and bioactive compounds, including lipids, proteins, and antioxidants. Particularly, the naked seeds of *Cucurbita pepo* var. *styriaca* have proved to yield both an edible oil showing anti-inflammatory properties in treating skin disorders and hydro-alcoholic extracts effective in inhibiting the growth of cancer cells. In this study, a detailed and extensive analysis of the eco-friendly alcoholic extract of the seeds of this variety was accomplished by using LC-HRMSMS techniques, with the main aim to broaden the knowledge on bioactive lipids other than the already reported fatty acids. The obtained results highlighted the occurrence of numerous compounds belonging to different classes of polar and neutral lipids, such as phospholipids, sphingolipids, glycolipids, acylglycerols, and oxylipins. Noteworthily, a significant presence of Cer-(EO)LCBs, i.e., Cer-EOS-type ceramides with different long chain base (LCB) and fatty acid composition, was detected, representing a real novelty for pumpkin. Additionally, a good number of multiflorane-type triterpenoids were detected, only some of which were previously reported in this plant. These findings highlight the nutraceutical value of these edible seeds.

## 1. Introduction

Since the early times of humanity, pumpkin plants, belonging to the genus *Cucurbita* (Cucurbitaceae family), have been cultivated as a major crop grown in tropical and temperate regions for their economic importance due to their nutritional value. They have also been used worldwide in traditional medicine for their anti-inflammatory, antioxidant, antiviral, and anti-diabetic properties [1,2].

Along with the pulp, the peel and seeds can be utilized for food purposes. In particular, pumpkin seeds, having a rich nutty flavor and chewy texture, once toasted and salted, are consumed in many countries directly as a snack, salad dressing, or breakfast cereal, served as an ingredient in bread or cakes, or used as additives in bakery industries [1,3]. They are an extraordinarily rich source of lipids (among which unsaturated fatty acids, i.e., UFAs), proteins, carbohydrates, fibers, and some essential micronutrients, as well as of bioactive compounds like phenolics, squalene, phytosterols, tocopherols (α, β, γ and δ), tocotrienols, carotenoids, and flavonoids. According to this, pumpkin seeds are gradually attracting attention in large-scale food, pharmaceutical, and cosmetic applications, changing their status nowadays from waste by-products of agro-industrial processes to super-seeds falling into the category of functional foods that may have the ability to establish a positive relationship between health and diet [1,2,3,4,5,6,7].

In this regard, available literature indicates that total edible oil extracted from pumpkin seeds may exert beneficial effects against benign prostatic hyperplasia (BPH) and could play a potential role in managing metabolic disorders, including diabetes-related conditions [8,9]. Further evidence, largely derived from seed oil fractions or preliminary experimental models, suggests that pumpkin seed oil could be associated with protective effects on cardiovascular health, particularly regarding hypertension and hypercholesterolemia [5,10]. Additionally, when applied topically, total oil extracts have shown potential in supporting skin homeostasis by demonstrating soothing, anti-inflammatory, wound-healing, and photo-protective properties [11,12,13].

A prominent example of these matrices is found in Austria, where the special Styrian pumpkin variety (*Cucurbita pepo* var. *styriaca*), characterized by ‘naked’ or hull-less seeds, has been cultivated for decades. The conditioned, green-colored oil obtained from its seeds is particularly renowned for its unique sensory and optical characteristics, such as a nutty aroma and a distinct dark-green color [9,14,15]. Recent studies investigating this total oil suggest potential anti-inflammatory properties upon topical application, exploring its possible use in the management of mild to moderate skin inflammation, including acne-related conditions [14,16,17]. Beyond the oil, eco-friendly hydro-alcoholic extracts of pumpkin seeds have also been investigated for their potential biological activities. Specifically, in vitro antiproliferative effects against prostate, breast, and colon cancer cell lines, as well as regulatory effects on hyperplastic cell models, have been reported, providing partial support for the traditional use of pumpkin seeds as a complementary approach in BPH management [18].

From a chemical-analytical point of view, the most studied lipid class for Styrian pumpkin seeds is represented by free fatty acids, with the predominance of essential fatty acids (FA) like linoleic ω-6 (LA or FA 18:2 n-6) and oleic ω-9 (OA or FA 18:1 n-9) with respect to palmitic (PA or FA 16:0) and α-linolenic ω-3 (ALA or FA 18:3 n-3) [9,14,15]. Limited research is currently available on other lipid classes, particularly polar lipids such as phospholipids (PLs), glycolipids (GLs), and sphingolipids (SLs). Beyond simply carrying essential fatty acids, polar lipids are fundamental to cell membrane integrity, cell signaling, regulation of inflammation, and skin barrier function. There is growing scientific interest in these lipids due to their nutritional importance and bioactivities, which include antioxidant, anti-inflammatory, antitumoral, immunomodulatory, and antimicrobial properties [19,20,21,22,23,24,25,26,27]. Thereby, analyzing these lipids could reveal functional health benefits of *Cucurbita pepo* var. *styriaca*, moving past standard fatty acid profiles. On the other hand, the technological advances in chromatography and mass spectrometry platforms allowed the establishment of analytical methods able to characterize a wide range of intact lipid molecules occurring in complex samples. Mass spectrometry-based lipidomics has recently advanced the structural and compositional characterization of native lipid profiles within the Cucurbitaceae family. For instance, a recent screening of eleven distinct *Cucurbita* cultivars has utilized an integrated platform combining gas chromatography (GC), GC-mass spectrometry (GC-MS), and ultra-high-performance liquid chromatography (UPLC) to map the baseline distribution of native fatty acids, sterols, triacylglycerols, and diacylglycerols [28]. To achieve deeper structural insights into specific lipid classes, complementary liquid chromatography modes have been successfully coupled with tandem mass spectrometry. Specifically, the orthogonal combination of reversed-phase (RPLC-MS/MS) and hydrophilic interaction liquid chromatography (HILIC-MS) has enabled the comprehensive profiling of polar lipids in *Cucurbita maxima* oily seeds, resolving over 180 phospholipid molecular species across eight distinct classes [29]. This high-throughput capability has been further exemplified in other Cucurbitaceae species, such as *Cucumis melo*, where untargeted UHPLC-MS/MS profiling has successfully annotated more than 2500 lipid molecules spanning 7 categories and 47 subclasses, underscoring a prominent structural and storage prevalence of glycerolipids and glycerophospholipids [30].

Therefore, this paper aims at gaining deep insights that can extend the state of the art on the lipid composition of Styrian pumpkin seeds, including, where possible, unusual subclasses, by analyzing its alcoholic extract obtained using environmentally friendly and low-toxicity food-grade ethyl alcohol, by applying a liquid chromatography coupled to high-resolution tandem mass spectrometry (LC-HRMSMS) method. In this way, highly diagnostic fragmentation spectra for each lipid class were generated, allowing us to assign the chemical formula to each product ion as well as to the precursor molecular ions, obtaining key structural information to explore the depth and breadth of lipid chemical diversity.

## 2. Materials and Methods

### 2.1. Plant Sample

The seeds of *Cucurbita pepo* var. *styriaca* (250 g), obtained from controlled organic cultivation of Styrian pumpkin in Austria, were purchased from Kräuterhaus Sanct Bernhard (Bad Ditzenbach, Germany) in September 2022. Samples were maintained at 4 °C during transport and stored at the same temperature.

### 2.2. Chemicals

Ethanol (absolute, ≥99.8%) for extraction was purchased from VWR International PBI S.r.l. (Milan, Italy). Acetonitrile (≥99.9%), water, and formic acid (≥99.8%) for LC-MS were purchased from VWR International PBI S.r.l. (Milan, Italy).

### 2.3. Sample Preparation Procedures

The pumpkin seeds were manually crushed. The entire procedure was performed using independent extraction replicates (n = 3). An amount of 5 g per replicate was immediately submitted to extraction by maceration with ethanol at room temperature and protected from light (50 mL × 3 days × 3 times, i.e., until the yield of the last extraction was <10%). In order to remove the non-extractable residue, each ethanolic extract was filtered through paper and concentrated under vacuum using a rotary evaporator. The method demonstrated high reproducibility, yielding a mean dry crude extract of 630 ± 4 mg, with an average percentage yield of 12.6% ± 0.08%. The resulting independent extracts were stored in air-tight glass vials at 4 °C for three days and subsequently analyzed separately under identical conditions.

### 2.4. UHPLC-Q-Orbitrap MS/MS Analysis

The ethanolic extracts of the seeds of *Cucurbita pepo* var. *styriaca* were analyzed by LC-ESI/HRMS, using a Thermo Ultimate RS 3000 ultrahigh-performance liquid chromatography (UHPLC) system coupled to a Q-Exactive high-resolution mass spectrometer equipped with a hybrid quadrupole-Orbitrap analyzer and HESI II heated electrospray ionization source (Thermo Fisher Scientific, Bremen, Germany), operating in positive and negative ionization modes. In accordance with our laboratory’s well-established chromatographic separation protocols for the analysis of polar lipids [19,23,31,32], separation was performed using a Symmetry 300 C-4 column (RP-4, 3.5 µm, 2.1 mm × 150 mm; Waters, Milford, MA, USA) held at 30 °C. The mobile phase consisted of 0.1% formic acid in water (*v*/*v*) as solvent A and 0.1% formic acid in acetonitrile (*v*/*v*) as solvent B, operating with a linear gradient from 30% to 95% B over 12 min, from 53% to 62% B over 9 min, from 62 to 95% B over 17 min, held at 95% B over 4 min, a flow rate of 0.2 mL/min. The autosampler was set to inject 8 μL of each ethanolic extract (0.5 mg/mL) in triplicate. In order to optimize analysis in both ionization modes, samples were run in separate batches for positive and negative polarities with dedicated HESI source parameters. Specifically, the spray voltage was set to 3.5 kV for positive mode and reduced to 2.5 kV for negative mode to prevent corona discharge. Capillary and probe heater temperatures, along with gas flows, were tailored independently for each batch to maximize desolvation efficiency based on polarity. The final parameters were capillary temperature 320 °C (positive) and 300 °C (negative); sheath gas 50 arbitrary units (a.u.); auxiliary gas 12.50 a.u. (positive) and 10 a.u. (negative); probe heater temperature 300 °C; and S-lens radio frequency (RF) voltage 50 a.u. The MS spectra were acquired in a mass range *m*/*z* 150–1400 with a resolution power of 70,000. To obtain HRMS/MS spectra, a “data dependent scan” experiment was performed, in which the first five intense ions in the HRMS spectrum were selected to be subjected to fragmentation, using a normalized collision energy (NCE) of 30 and a resolving power of 17,500. Xcalibur software (version 2.2) was used for instrument control, data acquisition, and data analysis. Metabolite identification was carried out through a manual comparative analysis of accurate precursor masses and MS/MS fragmentation pathways against peer-reviewed scientific literature. To validate the reliability of the qualitative profiling, the compounds were successfully annotated and included in the corresponding Table only if their specific mass spectrometric features and literature-matched criteria were consistently confirmed in all three independent extraction replicates. The validation thresholds were set as follows: an accurate mass error of <5 ppm for precursor ions and the presence of key diagnostic product ions matching literature-reported fragmentation pathways. System precision was established through outstanding retention time stability, with chromatographic behavior showing negligible variation (RSD < 1%) across all injections.

## 3. Results and Discussion

### 3.1. LC-HRMS/MS Analysis of the Styrian Pumpkin Seeds

In order to study the polar lipids of *C. pepo* var. *styriaca* seeds, ethanol was chosen as a safe, eco-friendly solvent, offering a sustainable alternative to toxic chloroform/methanol solvents. Although considered less effective in recovering highly hydrophobic lipids (such as neutral lipids and triacylglycerols), ethanol still extracted them efficiently enough for the study while offering a lower environmental impact. Additionally, ethanol’s high polarity served as a major advantage, effectively capturing highly interesting polar lipid classes, as highlighted by the LC-HRMS/MS analysis. This latter was carried out by using a UHPLC-Q-Exactive system in consideration of its sensitivity, high resolution, high mass accuracy, powerful product ion scanning capability, and ability to give in-depth lipid profile information, operating by both negative and positive electrospray ionizations, in consideration of the different structures and polarities of the lipid classes. Moreover, in order to reduce the strong interactions between the stationary phase and the hydrocarbon skeleton of higher molecular weight lipids, a RP-C4 column instead of a RP-C18 was used, allowing the shortening of retention time of less polar lipid classes, e.g., sphingolipids, and the complete separation of all polar lipid classes in a single chromatographic run. By studying the chromatographic behavior, accurate mass, and fragmentation pattern of each detected ion peak, and by comparing the acquired analytical data with those occurring in the literature, the molecular pool of the Styrian pumpkin variety could be defined as composed of metabolites mainly belonging to five distinct lipid classes, i.e., phospholipids, sphingolipids, glycolipids, oxylipins, and acylglycerols, along with specialized metabolites ascribable to the triterpenoid class (Figure S1).

#### 3.1.1. Phospholipids

The largest group among the compounds detectable in Styrian pumpkin seeds was undoubtedly represented by phospholipids, polar lipids being the main constituents of biological membranes and exerting important structural and functional properties [23,24]. Beyond their roles as structural components of cellular membranes, dietary phospholipids (PLs) possess significant nutritional, functional, and health-promoting relevance. Functionally, they act as natural emulsifiers that modulate lipid digestion and transport, while delivering essential fatty acids and choline directly for cellular signaling and membrane fluidity. Furthermore, evidence highlights their therapeutic potential in mitigating hepatic steatosis, improving cognitive function, and attenuating systemic inflammatory responses [33,34,35]. They are made up of several distinct moieties and different polar head groups; these latter define the different subclasses of phospholipids, in turn consisting of a mixture of many molecular species containing different combinations of fatty acids at the *sn*-1 and *sn*-2 positions on the glycerol backbone. The careful analysis of the tandem mass spectra allowed us to assign each compound to the correct PL subclass by giving information on the nature of the headgroup and allowing us to ascertain the main occurrence of phosphatidylcholines (PCs), phosphatidylethanolamines (PEs), and phosphatidylinositols (PIs) (Table 1). Moreover, phospholipids in which only one of the *sn*-1/*sn*-2 positions of glycerol is fatty acylated, i.e., the lyso-phospholipids (l-PL), were also detectable, all belonging to the previously mentioned PL subclasses, apart from one representative of the lyso-phosphatidic acid (l-PA) subclass (Table 1). Finally, two compounds assignable as *N*-acylglycerophosphatidylethanolamines (NA-GPEs), i.e., lipid compounds in which the only fatty acid is involved in an amide linkage with the amino head group of glycerophosphoethanolamine, along with three metabolites having the structure of phosphatidylethanols (PEths), i.e., phospholipids deriving from phosphatidic acids in which an ethyl group is located on the phosphate group [36], were identified (Table 1). As mentioned above, the tandem mass spectrometry technique represents a powerful tool for the lipid study as the fragmentation pathway of the components of each PL subclass allowed to discriminate them, even in the case, e.g., of l-PC/L-PE/NA-GPE and PC/PE, characterized by molecular formulae sharing the same heteroatomic composition (O_7_NP and O_8_NP, respectively) but yielding diagnostic product ions *via* typical neutral losses in tandem mass experiments. So, for example, the detection of a product ion at *m*/*z* 184 (C_5_H_15_O_4_NP) in the positive HRMSMS spectrum was promptly indicative of the phosphocholine head group, whereas the occurrence in negative tandem mass spectra of diagnostic product ions at *m*/*z* 214 and 196 (C_5_H_13_O_6_NP and C_5_H_11_O_5_NP, respectively) identified l-PE/PEs, corresponding to the glycero-phosphatidylethanolamine ion and to its mono-dehydrated form, respectively (Table 1). Instead, the occurrence in the negative HRMSMS spectrum of a main product ion originated from the [M-H]^−^ ion by neutral loss of the mono-dehydrated-glycerol moiety (74 Da, C_3_H_6_O_2_) and corresponding to the phosphorylated *N*-fatty amide head group ion, promptly identified the NA-GPEs (Table 1). Moreover, in the case of l-PI/PIs, the structural assignment was guided by the detection in the negative tandem mass spectrum of the diagnostic product ion at *m*/*z* 241 (C_6_H_10_O_8_P), corresponding to the dehydrated form of the inositol-phosphate. Finally, the occurrence in the negative HRMSMS spectrum of the product ion at *m*/*z* 152 (C_3_H_6_O_5_P), due to the mono-dehydrated glycerophosphate, allowed us to ascertain the presence of l-PA, while the detection of the product ion at *m*/*z* 125 (C_2_H_6_O_4_P), corresponding to a 2-hydroxyethyl phosphonate, was indicative of a PEth (Table 1).

Contemporarily, both negative and positive HRMSMS spectra allowed the assignment of the fatty acids composing l-PL/PL, by checking the occurrence of abundant R_x_COO^−^ fatty acid ions in the negative ion spectra and of product ions corresponding to the monodehydrated form of the monoacylated-glycerol unit in the positive ion spectra, respectively (Table 1). Moreover, the occurrence in this variety of *C. pepo* of a good number of l-PL/PL composed of oxidized fatty acids could be appreciated (Table 1). These latter could be assumed to be naturally present in the seeds of *Cucurbita pepo* var. *styriaca*, as a result of endogenous oxidative processes, rather than being considered as derived from artificial oxidation occurring during sample handling, extraction, and storage, as these procedures were conducted under controlled conditions for light, temperature, and storage atmosphere.

Comparison of the number of lipids belonging to each detected PLs subclass allowed us to estimate that phosphatidylcholines (PCs), phosphatidylethanolamines (PEs), and phosphatidylinositols (PIs), including their lyso-forms (l-PCs, l-PEs, and l-PIs), were the most represented subclasses in the pumpkin seed extract, with in particular the l-PC/PC subclass accounting for approximately 39% and both l-PE/PE and l-PI/PI for 26% each (Figure S2a). Moreover, it could be appreciated that in each of these three subclasses, the number of detected lyso-type compounds was significantly lower than that of the diacylated type (Figure S2b). It is noteworthy that, among the PLs with the highest peak intensities, PCs accounted for the largest number of species compared to PEs and PIs (Table 1).

By considering the composition in acyl chains of the different types of detected PLs, natural carriers of dietary FAs, a first difference among the main lyso-form subclasses in terms of composition in saturated and unsaturated FAs could be noted. In fact, l-PCs were mainly composed of unsaturated fatty acids, above all FA 18:2, in contrast to l-PEs, mainly made up of saturated FAs as 16:0 and 18:0, and l-PIs, having both the saturated and unsaturated species equally represented and composed of the same FAs so far considered (Figure S2c). Moreover, in the l-PCs were also comprised FAs in the oxygenated form (**3**, **4, 6**), not present in either l-PEs or in l-PIs, which instead included in their subclass a l-PI structure (**15**) composed of an unsaturated alkyl chain ether-linked rather than ester-linked to the glycerol core, a typology of etherPI described also in other plant seeds (Table 1) [37].

On the other hand, by considering the diacylated PLs, at least one fatty acid of each detected structure was unsaturated, and once again the FA 18:2 was the most represented one, followed by the FA 18:1, and only to a minimal extent by FAs 18:3 and 16:1 (Figure S2d). These results were in agreement with the literature on seed FA composition of the Styrian variety grown in Austria [9,14]. However, these reports considered either FAs released by transmethylation from total lipid classes and analyzed by GC-FID, or FAs composing TGs, rather than FAs occurring in PLs alone.

In any case, PEs resulted in the PLs compounds showing the greatest acylation ratio of unsaturated FAs *versus* saturated ones, followed by PCs and finally PIs, with the FA 16:0 being the most recurring saturated fatty acid. Furthermore, all the three main PL classes displayed the occurrence of more or less the same number of components in which one fatty acid was oxygenated, mainly being FA 18:2-O1 in PCs, FA 18:2-O2 in PEs and both FA types in PIs, even though PCs with no oxylipin were in higher number than those in which an oxidized acyl chain composed the structure (Table 1; Figure S2d).

#### 3.1.2. Sphingolipids

Among polar lipids occurring in the ethanol extract of Styrian pumpkin seeds, sphingolipids were the second group in terms of the number of components (Figure S1). SLs are an ubiquitous structural lipid class, characterized by a considerable structural variety, occurring in the biological membranes and performing additional essential functions as signaling molecules [21,22,23]. The basic building block of SLs is an amino-alcohol long-chain base (LCB), characterized by the presence of hydroxyl groups at C1 and C3 and an amino group at C2; it can vary on the basis of the length of the chain, commonly being C18, and its degree of hydroxylation and unsaturation. In the positive tandem mass spectra of Styrian pumpkin seed extract, five LCBs (**5**, **9**, **11**, **16**, **18**) could be detected, assigned by considering both their molecular formula and fragmentation pattern, which displayed main product ions generated by neutral loss of water molecules and CH_2_O moieties, useful to estimate the number of hydroxyl groups present in the sphingoid base (Table 2). Moreover, diagnostic product ions at *m*/*z* 106.0866 (C_4_H_12_O_2_N) and *m*/*z* 122.0814 (C_4_H_12_O_3_N), corresponding to the protonated forms of 2-aminobutane-1,3-diol and 3-aminobutane-1,2,4-triol, were observed in the positive-ion tandem mass spectra of compounds **9** and **11**, respectively. These ions enabled the identification of dihydrosphingosine (d18:0) and phytosphingosine (t18:0), respectively (Table 2). Another dihydroxylated LCB, likely *N*-acetylsphingosine (d18:1), and two trihydroxylated LCBs, likely dehydrophytosphingosine (t18:1) and C19-dehydrophytosphingosine (t19:1), could be additionally identified by considering, along with the others, the product ion generated by the neutral loss of a C_2_H_4_O moiety from the [M + H]^+^ ion of **16** and corresponding to an acetyl unit, and the product ion at *m*/*z* 74.0606 (C_3_H_8_ON), occurring in the trihydroxylated LCBs and likely corresponding to a protonated 2-aminopropanal formed by rearrangement of the hydroxyl group at C4 in aldehyde form, contemporary to the cleavage of the C3-C4 bond and neutral loss of a water molecule, respectively (Table 2).

By carefully analyzing both positive and negative tandem mass spectra, the occurrence of metabolites known as ceramides (Cers) could also be highlighted. These compounds are generated by *N*-acylation of the LCB amine group with fatty acids mainly composed of saturated or not 18 and 16 carbon chains and in some cases characterized by a hydroxyl group (hence FA h, instead of FA N, where N is for “non-hydroxylated”) which in plants is usually located at C-2 (**81**, **83**, **86**, **91**, **96**, **101**; Table 2).

**Figure 1:**
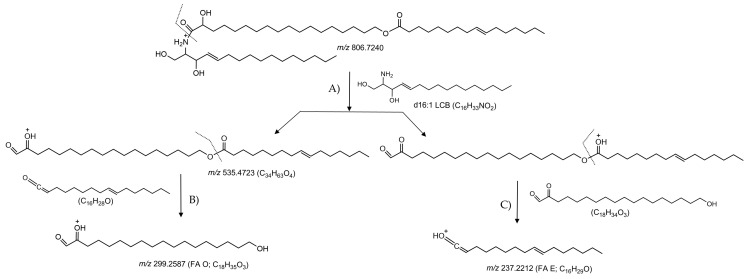
Proposed *de novo* MS/MS fragmentation pathway of the Cer-(EO)LCB subclass (case study compound **149**, precursor ion at *m/*z 806.7240). The scheme illustrates the manual structural elucidation based on HRMSMS spectra: (**A**) the primary cleavage driving the neutral loss of the intact d16:1 long-chain base (LCB) to yield the stable dual-acyl core product ion at *m/z* 535.4723 (C_34_H_63_O_4_); (**B**) the ester bond cleavage yielding the a,w-dihydroxylated inner acyl chain (FA O) as the diagnostic 3-oxygen product ion at *m/z* 299.2587 (C_18_H_35_O_3_); and (**C**) the ester bond cleavage yielding the outer acyl chain as a minor monodehydrated product ion at *m/z* 237.2212 (C_16_H_29_O). Note: The position of the double bond within the outer acyl chain (FA E) structure is tentative and illustrative only, as the exact double bond localization has not been assigned.

Interestingly, at later retention times, a large group of ceramides, showing in their tandem mass spectra product ions that suggested the occurrence of a second fatty acid unit in the structural core, could be detected (**117**–**118**, **120**, **122**, **126**, **129**, **131**–**133**, **137**–**155**; Table 2). In fact, the analysis of tandem mass spectra of these compounds allowed both the prompt ascertainment of the identity of the LCB groups—by observing the presence of a main product ion generated by neutral loss of the intact sphingoid base from the [M + H]^+^ ion—and the exclusion of the *O*-acylation of the second fatty acid on the LCB unit. For example, in the case of compound **149**, the occurrence in the tandem mass spectrum of the main product ion at *m*/*z* 535.4723, corresponding to the C_34_H_63_O_4_ elemental composition and originating from the neutral loss from the precursor ion of a structural moiety having the C_16_H_33_NO_2_ elemental composition, indicative of a d16:1 long chain base, could be observed. This finding along with the contemporary absence of product ions that could be formed by the removal of an acyl chain from the [M + H]^+^ ion, allowed to assign the ion at *m*/*z* 535.4723 as the dual-acyl core formed by the two fatty acids covalently linked to each other, thereby ruling out alternative structural isomers where the second FA was directly attached to the LCB (Table 2; Figure 1).

At the same time, by considering the elemental composition of this product ion, both the number of double bonds and overall the length of the carbon chain of the fatty acyl portion could be deduced (e.g., in the case of compound **149**, it was determined to be C34:1) (Table 2). Moreover, the careful analysis of the positive HRMSMS spectra of these compounds, highlighting the presence of a product ion having three oxygen atoms in its molecular formula, likely generated by the neutral loss of the monodehydrated form of the second fatty acyl chain, allowed to infer that the FA involved in the *N*-acylation with the LCB group of the ceramide had to be a FA holding an additional hydroxyl group in its structure (Table 2). Specifically, for the case study of compound **149**, it could be assumed that the product ion at *m*/*z* 299.2587 (C_18_H_35_O_3_) was generated by the cleavage of the ester bond (*via* neutral loss of a C_16_H_28_O moiety) and corresponded to the dihydroxylated form of the octadecanoic acid, i.e., a α-hydroxylated FA (FA h, inner acyl-chain) with an additional hydroxyl group, native to its structure (Table 2; Figure 1). Furthermore, the detection of a minor product ion at *m*/*z* 237.2212 (C_16_H_29_O) allowed the assignment of the second fatty acid (outer acyl-chain) under the well-stabilized form of a monodehydrated acyl-chain generated during the ester bond cleavage (Table 2; Figure 1). Some literature reports describe the occurrence in seeds of plants such as sesame and *Camellia oleifera* of a typology of ceramides known as Cer-EOS (**Cer**amide by **E**sterification of the **O**mega-hydroxyl group of fatty acid linked on **S**phingosine), i.e., ceramides in which the fatty acid *N*-acylated to the LCB sphingosine has an ω-hydroxyl group (hence FA O) involved in the esterification of a second fatty acid (hence FA E). Considering this, a similar structural composition for the metabolites identified in Styrian pumpkin seed extract [38,39] could be tentatively suggested. Moreover, the occurrence in the tandem mass spectra of product ions ascribable to consecutive fragmentations involving both fatty acyl chains confirmed this hypothesis (Table 2). These findings are noteworthy considering that this is the first study describing the occurrence of this compound class in pumpkin. It is well-known that a deficiency of ceramides, essential components of the lipid skin barrier, may account, at least in part, for the dysfunction of the skin stratum corneum associated with aging and other inflammatory skin disorders (e.g., atopic dermatitis, psoriasis, ichthyoses). In fact, topical application of ceramide-based formulations as well as dietary ceramide-based supplementations have been proven as treatments able to restore skin-ceramide levels and lipid organization in the stratum corneum, by improving the epidermal barrier functionality in damaged skin, showing positive effects on skin-moisturizing and skin barrier recovery [40,41,42,43,44]. By considering the peculiar structural features of Cer-EOS molecules, it could be postulated that they have a potential structure–activity relationship in determining physical skin-barrier restoration, limiting trans-epidermal water loss. The ultra-long carbon chain, resulting from the mutual esterification of two fatty acids, may allow Cer-EOS molecules to act as a “bridge” between adjacent lipid lamellae in the stratum corneum. This arrangement would promote the organization of lipids into a long-periodicity phase, which is essential for skin impermeability. Furthermore, the presence of unsaturation on the terminal acyl chain could introduce molecular kinks, creating fluid microdomains within an otherwise rigid crystalline matrix. This structural alternation between crystalline and fluid domains may physically prevent water evaporation and shield the skin from external pathogens. Thereby, a potential role for these molecules in the treatment of skin inflammation could be hypothesized, in line with literature evidence ascribing similar anti-inflammatory properties to Styrian pumpkin seed variety, although it must be noted that existing studies investigated the oil matrix [16,17]. Finally, two metabolites belonging to the subclass of hexosylceramides (HexCers), glycosylation products of the primary hydroxyl group of the LCB unit, usually with glucose or galactose, were detectable in HRMSMS spectra of both polarity by observing, along with the product ions generated by fragmentation pathway involving the LCB and the FA moieties, the presence of product ions formed by neutral loss of C_6_H_10_O_5_ and C_6_H_12_O_6_ units, corresponding to the hexose group in the monodehydrated and whole form, respectively (**62**, **66**; Table 2).

Evaluation of the number of species within the different SL structural groups revealed that Cer-(EO)LCB—so named due to their varying LCB composition—was the most represented subclass, both numerically and in terms of peak intensity (Figure S3a; Table 2). In particular, by considering the type and the number of sphingoid bases composing SL structures, undoubtedly Cer-(EO)LCBs showed the highest variability (Figure S3b). Notably, the most occurring LCB was the d16:1, likely a C16-sphingosine, rather than the C18-sphingosine, immediately followed, but to a lesser extent, by the LCB d18:2, likely sphingadienine, and the trihydroxylated LCB t18:2, previously described in the fungus *Cordyceps sinensis* [25], and the LCB t18:1, likely dehydrophitosphingosine (Figure S3b). Interesting was also the evaluation of the type of FAs *N*-acylating the sphingoid bases of SLs (Figure S3c). In fact, if Cers showed a predominance of N-type FAs with respect to HexCers, the FAs composing SLs of both subclasses were characterized only by acyl chains with an even number of carbon atoms, in contrast to those belonging to Cer-(EO)LCB subclass, mainly characterized by long acyl chains mostly having an odd number of carbon atoms, being FA O21:2 the most numerous. On the contrary, the E-type FAs were chemically characterized by shorter acyl chains, mostly with an even number of carbon atoms, with FA E16:1 being the most represented along with FA E18:2 (Figure S3d).

#### 3.1.3. Acylglycerols

The third class in order of number of components detected in the ethanolic extract of Styrian pumpkin seeds was that of acylglycerols (AGs) (Figure S1; Table 3), structurally made up of a glycerol core esterified with one to three fatty acids to form mono-, di-, and triacylglycerols (MGs, DGs, and TGs), respectively. In plants, diacylglycerols are intermediates in the biosynthesis of triacylglycerols and other glycerolipids, including many phospholipids and mono- and digalactosyldiacylglycerols, while TGs are important high-energy compounds and a source of structural fatty acids, providing energy and carbon skeletons for seed germination and seedling development [45]. Analogously, triacylglycerols are the main dietary source of energy and essential fatty acids in humans [46]. Their fatty acid composition strongly influences cardiovascular and metabolic health, particularly the balance between saturated and unsaturated fatty acids [47]. Mono- and diacylglycerols are important intermediates in lipid digestion and enhance intestinal absorption of lipids and lipid-soluble vitamins [46]. Diets rich in unsaturated triacylglycerols have been associated with improved lipid profiles and reduced risk of cardiovascular disease [47,48].

The structural features of these lipids were promptly highlighted by observing in the tandem mass spectra acquired in positive ion mode the presence of product ions generated by neutral loss of one or two whole FAs, the first occurring both in diacylglycerols (DGs) and in triacylglycerols (TGs) HRMSMS spectra, the second ones detectable only in fragmentation spectra of TGs (Table 3). In addition to product ions corresponding to the monodehydrated FAs, allowing us to assign their identity by analyzing the HRMSMS spectra of both acylglycerol subclasses, the fragmentation pattern of DGs was also characterized by diagnostic product ions formed from the [M + H]^+^ ion by neutral loss of a water molecule (Table 3). Moreover, the neutral loss of two FA units in TGs allowed the detection of product ions corresponding to the didehydrated form of the monoacylated glycerol core (Table 3). Interestingly, the mass spectrometric data allowed to identify in Styrian pumpkin seed extract the occurrence of two metabolites belonging to a third subclass, namely monoacylglycerol ethers (MGEs), characterized by a different form of functionalization of the glycerol core in which an alkyl substituent is ether-bound at one glycerol oxygen and an acyl substituent is *O*-esterified at another glycerol position, with usually the location of etherification being at *sn-*1-position, and the *O*-acylation occurring at the *sn-*2-position. This type of naturally occurring lipid is considered a functional lipid with well-recognized health benefits, showing a positive correlation between dietary intake and treatment and prevention of several diseases, including obesity, diabetes, inflammation, and cancer [37,49]. The analysis of tandem mass spectra showed the product ion corresponding to the monodehydrated acylic portion, along with the product ion corresponding to the protonated alcohol involved in the ether-linkage with the glycerol unit. These data, along with the molecular formula ascribable to these lipid compounds, displaying four oxygen atoms instead of five, as in the case of DGs, supported this structural hypothesis (**87**, **93**; Table 3). Among the three acylglycerol subclasses, MGE was the least represented, while the TG subclass was the most numerous and featured the compounds with the highest peak intensities (Figure S4a; Table 3). The analysis of FA composition revealed that DGs were mainly composed of FA 18:1, in contrast to TGs, which showed a predominance of FA 18:2, the only one present in MGEs, and of FA 18:3 (Figure S4b). Notably, TGs were the only acylglycerol subclass displaying oxidized FAs on the glycerol core.

#### 3.1.4. Triterpenoids

The analysis of the mass spectrometric data allowed us to ascertain the occurrence in the ethanolic extract of the Styrian pumpkin seeds of specialized metabolites belonging to the triterpenoid class, a large group of natural products, derived biosynthetically from the acyclic C-30 hydrocarbon squalene, most of which show tetracyclic and pentacyclic skeletons. Known for their roles in plant development processes and defense response, triterpenoids are characterized by a wide range of biological activities, including anti-inflammatory, anti-proliferative, antidiabetic, hepatoprotective, antimicrobial, antimycotic, analgesic, immunomodulatory, and cardiotonic [50,51].

The analysis of the tandem mass spectrum of the [M + H]^+^ ion at *m*/*z* 682.4452 (**59**), characterized by the molecular formula C_44_H_59_O_5_N, highlighted the occurrence of a diagnostic product ion at *m*/*z* 527.3889 (C_37_H_51_O_2_), originated by contemporary neutral losses of a water molecule and of a C_7_H_7_O_2_N unit corresponding to an aminobenzoic acid, along with a product ion at *m*/*z* 405.3508 (C_30_H_45_) formed by neutral loss of a benzoic acid unit (Table 4). This fragmentation pattern and the literature data allowed us to identify the C30 skeleton as that of multiflorane-type triterpenoids, in agreement with that previously reported by [52] that, in the seeds of *C. pepo*, described the occurrence of an *O*-*p*-aminobenzoyl-*O*-benzoyl-multifloren-triol derivative along with two other specialized metabolites of this class, the *O*-aminobenzoyl-*O*-benzoyl-multiflora-dien-diol and *O*-aminobenzoyl-multiflora-dien-diol, which could correspond respectively to compound **99**, the dehydrated form of **59**, and compound **60**, the debenzoylated form of **99** (Table 4).

By considering the mass spectrometric fragmentation pathway described above, the structures of four new multiflorane derivatives could be tentatively assigned to compound **36**, likely corresponding to the debenzoylated form of **59**, i.e., the *O*-aminobenzoyl-multifloren-triol derivative, to compound **38**, likely the monoesterified form of bryonolic acid with an aminobenzoic acid, to compound **42**, likely corresponding to the esterified form of **36** with another aminobenzoic acid, and to compound **45**, likely corresponding to the acetylated form of **36**, i.e., the *O*-aminobenzoyl-*O*-acetyl-multifloren-triol, respectively (Table 4).

Analogously, the structure of the other four triterpenoids could be assigned as that of two multiflora-dien-diol derivatives (**40** and **43**), likely karounidiol and its isomer isokarounidiol, a multiflora-trien-diol derivative (**39**), likely dehydrokarounidiol, and an oxo-multiflorenoic acid derivative (**41**), likely bryononic acid, all of them already described in some Cucurbitaceae species [53,54,55,56] (Table 4) but never reported before in pumpkin (Table 1). The finding of multiflorane-type triterpenoids in the seeds of *C. pepo* var. *styriaca* is noteworthy and might suggest a potential basis for their medicinal and nutraceutical interest, considering the melanogenesis inhibitory, cancer-preventing, and antioxidant properties previously described for this chemical class [51,57]. In the absence of targeted bioassays, any direct involvement of the identified compounds remains speculative; however, it could be tentatively proposed that the specific core arrangement of these multiflorane derivatives—including oxygenation patterns at C-3, C-7, and C-29, and esterification with aromatic groups like *p*-aminobenzoic acid (PABA) or benzoyl moieties—could influence their radical scavenging capacity or interact with intracellular signaling pathways (e.g., NF-κB/MAPK). Furthermore, one might speculate that the rigid, hydrophobic planar core of the multiflorane skeleton could interact with the fluid domains of the stratum corneum lipid matrix. By theoretically mimicking cholesterol, these compounds might assist in stabilizing compromised epidermal membranes, modulating membrane fluidity and protecting keratinocyte integrity together with Cer-EOS.

#### 3.1.5. Oxylipins

The analysis of HRMSMS data acquired in negative ionization mode concurred to identify as fourth lipid class, in order of detected components, that of oxylipins, bioactive lipid mediators produced from the polyunsaturated fatty acid (PUFA) oxidative metabolism, differing each other for the degree of unsaturation and oxygenation of the acyl chains, and described as involved in in vivo inflammatory cascades, pain perception, and skin barrier integrity [23,57]. The detection in tandem mass spectra of main product ions originated by consecutive neutral losses of water molecules allowed to promptly identify in the structure both the occurrence and the number of hydroxy groups, whose presence could be furthermore supported by the detection of diagnostic product ions formed by a CHOH→CHO rearrangement involving the hydroxyl groups and generating shortened aldehydic acyl chains from the end-part or the head-part of the oxylipin (Table 5). On the other hand, by considering the elution order trend, showing the earlier elution for trihydroxylated with respect to di- and monohydroxylated oxylipins such as for diunsaturated oxylipins with respect to the monounsaturated ones, the identification at delayed retention times of di- and mono-unsaturated oxo-forms could be supposed (**31**, **33**), moreover supported by the fragmentation pattern, showing, e.g., as main product ion that originated by neutral loss of a CO_2_ molecule instead of those formed by dehydroxylation (Table 5) [19,23,31,32]. To our knowledge, this is the first study reporting these metabolites in Styrian pumpkin seeds, since only phytoprostane-type oxylipins have been described for this variety so far [14].

#### 3.1.6. Glycolipids

The least represented class of lipids in the extract of Styrian pumpkin seed was that of glycolipids (GLs), which included only three compounds (Table 5). Glycolipids are the major components of photosynthetic membranes and have been reported to display antiviral, antitumor, and anti-inflammatory activities [19,23,31,58]. Structurally, they are characterized by different combinations of fatty acids esterified at the *sn*-1 and *sn*-2 positions of the glycerol unit, but differing in the nature of the sugar moiety linked at the *sn*-3 position, so composing different subclasses, such as that of digalactosyldiacylglicerols (DGDGs) and the sulfoquinovosyldiacylglycerols (SQDGs), made up of galactose or sulfoquinovose, respectively (Table 5). Thereby, the metabolites belonging to these two GL subclasses can be easily distinguished already on the basis of their different molecular formulae, that, e.g., include a sulfur atom in the case of SQDG, and of course by their fragmentation pattern. So, the molecular formula C_39_H_74_O_12_S detectable in Styrian pumpkin seed (**37**, Table 5) could be assigned to an SQDG-type metabolite by checking the presence in the HRMSMS spectrum of the product anion at *m*/*z* 225, originated from the cleavage of glycerol-sugar ether linkage and resulting in the formation of an epoxydic bridge between carbon 1 and 2 of the sulfoquinovosylic ring. Analogously, the assignment of the two detected DGDGs (**75**, **79**) could be supported by the finding in the tandem mass spectra of the product cation generated by neutral loss of the monodehydrated form of galactose, along with those formed by neutral loss of the whole acyl chain (Table 5). By considering the acyl chain types composing the structures of identified GLs, the predominance of FA 18:2 could be observed in DGDGs, while the only found SQDG was formed by shorter saturated acyl chains, i.e., 14:0 and 16:0 (Table 5).

## 4. Conclusions

In conclusion, the results provided in this study allowed us to broaden the knowledge on lipid composition of the edible seeds of the Styrian pumpkin variety, in particular highlighting the presence of different lipid classes, ranging from the more polar ones, such as phospholipids, sphingolipids and glycolipids, to the neutral ones, such as acylglycerols, up to including low molecular weight lipids, such as oxylipins.

Particularly interesting is the identification of the Cer-EOS-type ceramide lipid subclass, described here for the first time in pumpkin and characterized by compounds displaying some of the highest peak intensities among all the identified lipid classes. The presence of these ceramides could enhance the functional value of the edible seeds of the Styrian pumpkin variety, suggesting their potential interest as a dietary supplement. Although future targeted bioassays are required to confirm any direct health benefits, it might tentatively be hypothesized that these compounds could play a role in supporting the management of skin inflammation. This assumption would align with literature reports on the anti-inflammatory properties of the Styrian pumpkin seeds, although a distinction must be made since those studies focus on the oil matrix rather than an alcoholic extract.

Moreover, it is noteworthy that this method of analysis allowed us to ascertain the occurrence in the seeds of *Cucurbita pepo* var. *styriaca* of specialized metabolites such as the multiflorane-type triterpenoids, some of which have never been described before in pumpkin. Representing a particular chemical class of metabolites with melanogenesis-inhibitory, cancer-preventing, and antioxidant activities reported in the literature, their occurrence further highlights the chemical complexity and potential nutraceutical value of these edible seeds.

In conclusion, the results of this chemical profiling study can be considered a preliminary baseline that opens promising perspectives for future investigations focused on the absolute quantification and biological activity verification of the newly identified classes of lipids and specialized metabolites.

## Figures and Tables

**Table 1:** Phospholipids tentatively identified in the EtOH extract of *C. pepo* var. *styriaca* seeds.

Phospholipids												
N°	Compound	R_t_ (min)	Molecular Formula	[M-H]^−^	[(M + HCO_2_H)-H]^−^	RDB	ppm	[M + H]^+^	RDB	ppm	HRMS/MS	MS Peak Intensity (NL)
**3**	l-PC (18:2-O2)	8.51	C_26_H_50_O_9_NP		596.3220	3.5	3.23				293.2121 (C_18_H_29_O_3_); 242.0790 (C_7_H_17_O_6_NP); 224.0701 (C_7_H_15_O_5_NP); 78.9577(O_3_P)	1.79 × 10^5^
								552.3300	2.5	0.66	184.0734 (C_5_H_15_O_4_NP); 125.0004 (C_2_H_6_O_4_P)	2.18 × 10^5^
**4**	l-PC (18:2-O)	9.18	C_26_H_50_O_8_NP		580.3265	3.5	3.49				520.3049 (C_25_H_47_O_8_NP); 295.2278 (C_18_H_31_O_3_); 224.0692 (C_7_H_15_O_5_NP)	1.09 × 10^5^
								536.3351	2.5	0.85	184.0736 (C_5_H_15_O_4_NP); 125.0003 (C_2_H_6_O_4_P)	1.10 × 10^5^
**6**	l-PC (18:0-O2)	10.08	C_26_H_52_O_9_NP		598.3370	2.5	3.18				295.2283 (C_18_H_31_O_3_)	4.31 × 10^4^
								554.3459	1.5	1.27	184.0736 (C_5_H_15_O_4_NP)	5.85 × 10^4^
**7**	NA-GPE (18:3)	10.24	C_23_H_42_O_7_NP	474.2631		4.5	3.38				400.2259 (C_20_H_35_O_5_NP); 171.0048 (C_3_H_8_O_6_P); 152.9948 (C_3_H_6_O_5_P)	4.46 × 10^5^
								476.2774	3.5	0.58	304.2640 (C_20_H_34_ON)	5.45 × 10^5^
**8**	l-PC (16:2)	10.38	C_24_H_46_O_7_NP		536.2994	3.5	2.02				251.2015 (C_16_H_27_O_2_); 224.0693 (C_7_H_15_O_5_NP)	4.21 × 10^4^
								492.3088	2.5	0.76	184.0737(C_5_H_15_O_4_NP)	5.76 × 10^4^
**10**	l-PC (14:0)	10.70	C_22_H_46_O_7_NP		512.2996	1.5	2.47				227.2012 (C_14_H_27_O_2_); 224.0689 (C_7_H_15_O_5_NP)	6.37 × 10^4^
								468.3087	0.5	0.47	184.0737 (C_5_H_15_O_4_NP)	1.04 × 10^5^
**12**	l-PI (18:2)	11.24	C_27_H_49_O_12_P	595.2896		4.5	3.05				315.0512 (C_9_H_16_O_10_P); 279.2344 (C_18_H_31_O_2_); 241.0115 (C_6_H_10_O_8_P); 152.9948 (C_3_H_6_O_5_P)	1.01 × 10^6^
								597.3036	3.5	0.35	337.2735 (C_21_H_37_O_3_)	5.73 × 10^4^
**13**	NA-GPE (18:2)	11.55	C_23_H_44_O_7_NP	476.2780		3.5	1.79				402.2407 (C_20_H_37_O_5_NP); 152.9947 (C_3_H_6_O_5_P)	1.51 × 10^6^
								478.2931	2.5	0.64	306.2791 (C_20_H_36_ON)	3.11 × 10^6^
**14**	l-PI (18:2)	11.82	C_27_H_49_O_12_P	595.2888		4.5	1.63				415.2245 (C_21_H_36_O_6_P); 315.0484 (C_9_H_16_O_10_P); 279.2326 (C_18_H_31_O_2_); 241.0114 (C_6_H_10_O_8_P); 152.9947 (C_3_H_6_O_5_P); 78.9577 (O_3_P)	4.24 × 10^6^
								597.3033	3.5	−1.08	337.2736 (C_21_H_37_O_3_); 263.2368 (C_18_H_31_O); 155.0104 (C_3_H_8_O_5_P)	3.01 × 10^5^
**15**	Ether-l-PI (O-16:2)	11.93	C_25_H_47_O_11_P					555.2925	2.5	−0.61	313.2737 (C_19_H_37_O_3_); 239.2365 (C_16_H_31_O); 155.0104 (C_3_H_8_O_5_P)	5.22 × 10^5^
**17**	l-PC (18:3)	12.13	C_26_H_48_O_7_NP		562.3147	4.5	1.34				277.2168 (C_18_H_29_O_2_); 224.0689 (C_7_H_15_O_5_NP); 168.0427 (C_4_H_11_O_4_NP)	5.98 × 10^5^
								518.3242	3.5	0.08	500.3164 (C_26_H_47_O_6_NP); 184.0736(C_5_H_15_O_4_NP)	8.62 × 10^5^
**19**	l-PI (16:0)	12.52	C_25_H_49_O_12_P	571.2887		2.5	1.15				391.2247 (C_19_H_36_O_6_P); 315.0486 (C_9_H_16_O_10_P); 255.2326 (C_16_H_31_O_2_); 241.0113 (C_6_H_10_O_8_P); 152.9947 (C_3_H_6_O_5_P); 78.9577 (O_3_P)	1.98 × 10^6^
								573.3033	1.5	−0.28	313.2737 (C_19_H_37_O_3_); 155.0104 (C_3_H_8_O_5_P)	1.24 × 10^5^
**20**	l-PE (18:2)	12.89	C_23_H_44_O_7_NP	476.2776		3.5	0.70				279.2327 (C_18_H_31_O_2_); 214.0479 (C_5_H_13_O_6_NP); 196.0373 (C_5_H_11_O_5_NP)	1.36 × 10^7^
								478.2921	2.5	−1.47	337.2733 (C_21_H_37_O_3_)	1.31 × 10^7^
**21**	l-PE (16:0)	12.93	C_21_H_44_O_7_NP	452.2778		1.5	1.47				255.2340 (C_16_H_31_O_2_); 214.0479 (C_5_H_13_O_6_NP); 196.0369 (C_5_H_11_O_5_NP)	9.49 × 10^5^
								454.2927	0.5	−1.55	313.2736 (C_19_H_37_O_3_)	7.78 × 10^5^
**22**	l-PC (18:2)	12.98	C_26_H_50_O_7_NP		564.3300	3.5	0.75				504.3067 (C_25_H_47_O_7_NP); 279.2326 (C_18_H_31_O_2_); 242.0795 (C_7_H_17_O_6_NP); 78.9575 (O_3_P)	7.02 × 10^6^
								520.3394	2.5	−1.71	502.3277 (C_26_H_49_O_6_NP); 337.2740 (C_21_H_37_O_3_); 184.0734 (C_5_H_15_O_4_NP)	8.33 × 10^6^
**23**	l-PE (16:0)	13.35	C_21_H_44_O_7_NP	452.2776		1.5	0.94				255.2326 (C_16_H_31_O_2_); 214.0479 (C_5_H_13_O6NP); 196.0371 (C_5_H_11_O_5_NP)	6.93 × 10^6^
								454.2922	0.5	−1.33	313.2737 (C_19_H_37_O_3_)	7.04 × 10^6^
**24**	l-PC (18:2)	13.38	C_26_H_50_O_7_NP		564.3295	3.5	−0.12				504.3097 (C_25_H_47_O_7_NP); 279.2324 (C_18_H_31_O_2_); 224.0688 (C_7_H_15_O_5_NP); 78.9577 (O_3_P)	3.27 × 10^7^
								520.3394	2.5	−1.71	502.3288 (C_26_H_49_O_6_NP); 337.2730 (C_21_H_37_O_3_); 184.0735 (C_5_H_15_O_4_NP)	4.78 × 10^7^
**26**	l-PC (16:0)	13.89	C_24_H_50_O_7_NP		540.3302	1.5	1.12				480.3087 (C_23_H_47_O_7_NP); 255.2325 (C_16_H_31_O_2_); 242.0793 (C_7_H_17_O_6_NP); 224.0685 (C_7_H_15_O_5_NP); 152.9947 (C_3_H_6_O_5_P); 78.9575 (O_3_P)	9.95 × 10^6^
								496.3397	0.5	−1.25	478.3302 (C_24_H_49_O_6_NP); 313.2736 (C_19_H_37_O_3_); 184.0733 (C_5_H_15_O_4_NP)	1.02 × 10^7^
**27**	l-PE (18:1)	14.08	C_23_H_46_O_7_NP	478.2939		2.5	1.14				281.2483 (C_18_H_33_O_2_); 196.0371 (C_5_H_11_O_5_NP)	1.66 × 10^6^
								480.3087	1.5	0.59	339.2893 (C_21_H_39_O_3_)	1.77 × 10^6^
**28**	l-PA (18:2)	14.23	C_21_H_39_O_7_P	433.2360		3.5	2.36				279.2328 (C_18_H_31_O_2_); 171.0055 (C_3_H_8_O_6_P); 152.9947 (C_3_H_6_O_5_P); 78.9577(O_3_P)	1.13 × 10^6^
**30**	l-PC (18:1)	14.37	C_26_H_54_O_7_NP		566.3465	2.5	1.24				506.3265 (C_25_H_49_O_7_NP); 281.2482 (C_18_H_33_O_2_); 242.0793 (C_7_H_17_O_6_NP); 78.9574 (O_3_P)	8.52 × 10^6^
								522.3558	1.5	−0.25	504.3439 (C_26_H_51_O_6_NP); 339.2877 (C_21_H_39_O_3_); 184.0733 (C_5_H_15_O_4_NP)	1.36 × 10^7^
**32**	l-PI (18:0)	14.74	C_27_H_53_O_12_P	599.3207		2.5	1.43				419.2583 (C_21_H_40_O_6_P); 315.0483 (C_9_H_16_O_10_P); 283.2641 (C_18_H_35_O_2_); 241.0115 (C_6_H_10_O_8_P); 152.9948 (C_3_H_6_O_5_P); 78.9577 (O_3_P)	7.63 × 10^5^
								601.3342	1.5	−0.95	341.3053 (C_21_H_41_O_3_); 155.0106 (C_3_H_8_O_5_P)	6.65 × 10^4^
**34**	l-PE (18:0)	15.71	C_23_H_48_O_7_NP	480.3095		1.5	2.23				283.2640 (C_18_H_36_O_2_); 196.0372 (C_5_H_11_O_5_NP); 140.0107 (C_2_H_7_O_4_NP)	3.95 × 10^5^
								482.3248	0.5	0.21	341.3053 (C_21_H_41_O_3_)	4.76 × 10^5^
**35**	l-PC (18:0)	15.95	C_26_H_54_O_7_NP		568.3630	1.5	2.70				508.3408 (C_25_H_51_O_7_NP); 283.2639 (C_18_H_35_O_2_); 242.0801 (C_7_H_17_O_6_NP); 224.0689 (C_7_H_15_O_5_NP); 152.9945 (C_3_H_6_O_5_P)	2.14 × 10^6^
								524.3716	0.5	0.04	184.0735 (C_5_H_15_O_4_NP)	4.04 × 10^6^
**44**	PI (18:2; 18:2-O2)	21.12	C_45_H_79_O_15_P	889.5097		7.5	2.71				577.2767 (C_27_H_46_O_11_P); 415.2258 (C_21_H_36_O_6_P); 315.0500 (C_9_H_16_O_10_P); 293.2120 (C_18_H_29_O_3_); 279.2326 (C_18_H_31_O_2_); 241.0115 (C_6_H_10_O_8_P); 223.0007 (C_6_H_8_O_7_P); 152.9948 (C_3_H_6_O_5_P)	6.55 × 10^5^
**46**	PI (16:0; 18:2-O1)	21.37	C_43_H_79_O_14_P	849.5154		5.5	3.54				553.2763 (C_25_H_46_O_11_P); 431.2180 (C_21_H_36_O_7_P); 391.2253 (C_19_H_36_O_6_P); 295.2272 (C_18_H_31_O_3_); 255.2326 (C_18_H_31_O_3_); 241.0116 (C_6_H_10_O_8_P); 152.9961 (C_3_H_6_O_5_P)	3.69 × 10^5^
**47**	PI (16:0; 18:2-O2)	21.70	C_43_H_79_O_15_P	865.5101		5.5	3.28				571.2884 (C_25_H_48_O_12_P); 553.2773 (C_25_H_46_O_11_P); 409.2358 (C_19_H_38_O_7_P); 391.2256 (C_19_H_36_O_6_P); 297.0372 (C_9_H_14_O_9_P); 255.2328 (C_16_H_31_O_2_); 241.0122 (C_6_H_10_O_8_P); 223.0013 (C_6_H_8_O_7_P); 152.9949 (C_3_H_6_O_5_P)	2.07 × 10^6^
**48**	PI (16:0; 18:3-O1)	22.31	C_43_H_77_O_14_P	847.4987		6.5	2.29				391.2270 (C_19_H_36_O_6_P); 293.2120 (C_18_H_29_O_3_); 255.2327 (C_16_H_31_O_2_); 241.0121 (C_6_H_10_O_8_P); 152.9949 (C_3_H_6_O_5_P)	3.88 × 10^4^
**49**	PI (18:2; 18:2-O1)	22.38	C_45_H_79_O_14_P	873.5130		7.5	0.72				593.2735 (C_27_H_46_O_12_P); 577.2795 (C_27_H_46_O_11_P); 431.2203 (C_21_H_36_O_7_P); 415.2245 (C_21_H_36_O_6_P); 315.0495 (C_9_H_16_O_10_P); 295.22278 (C_18_H_31_O_3_); 279.2328 (C_18_H_31_O_2_); 241.0116 (C_6_H_10_O_8_P); 152.9947 (C_3_H_6_O_5_P)	5.11 × 10^5^
**50**	PC (18:2-O1; 18:2-O1)	22.48	C_44_H_80_O_10_NP		858.5515	6.5	2.82				295.2279 (C_18_H_31_O_3_); 277.2178 (C_18_H_29_O_2_); 224.0689 (C_7_H_15_O_5_NP)	1.68 × 10^5^
								814.5596	5.5	−0.22	184.0735 (C_5_H_15_O_4_NP); 86.0969 (C_5_H_12_N)	
**51**	PE (18:2; 18:2-O2)	22.52	C_41_H_74_O_10_NP	770.4989		6.5	2.19				311.2234 (C_18_H_31_O_4_); 293.2121 (C_18_H_29_O_3_); 279.2327 (C_18_H_31_O_2_); 275.2025 (C_18_H_27_O_2_); 140.0108 (C_2_H_7_O_4_NP)	2.43 × 10^6^
**52**	PI (16:0; 18:2-O1)	23.08	C_43_H_79_O_14_P	849.5146		5.5	2.03				553.2744 (C_25_H_46_O_11_P); 391.2261 (C_19_H_36_O_6_P); 295.2277 (C_18_H_31_O_3_); 255.2325 (C_16_H_31_O_2_); 241.0116 (C_6_H_10_O_8_P); 223.0011 (C_6_H_8_O_7_P); 152.9948 (C_3_H_6_O_5_P)	7.01 × 10^5^
**53**	PE (18:2; 18:3-O1)	23.21	C_41_H_72_O_9_NP	752.4875		7.5	1.93				293.2119 (C_18_H_29_O_3_); 279.2336 (C_18_H_31_O_2_)	3.27 × 10^4^
**54**	PE (16:0; 18:2-O2)	23.42	C_39_H_74_O_10_NP	746.4987		4.5	1.94				452.2794 (C_21_H_43_O_7_NP); 434.2668 (C_21_H_41_O_6_NP); 311.2246 (C_18_H_31_O_4_); 293.2121 (C_18_H_29_O_3_); 255.2326 (C_16_H_31_O_2_); 140.0108 (C_2_H_7_O_4_NP)	2.11 × 10^6^
**55**	PE (18:2; 18:1-O2)	23.73	C_41_H_76_O_10_NP	772.5142		5.5	2.47				295.2281 (C_18_H_31_O_3_); 279.2332 (C_18_H_31_O_2_); 140.0107 (C_2_H_7_O_4_NP)	1.94 × 10^5^
**56**	PE (18:1; 18:2-O2)	23.80	C_41_H_76_O_10_NP	772.5139		5.5	2.08				293.2118 (C_18_H_29_O_3_); 281.2484 (C_18_H_33_O_2_); 140.0105 (C_2_H_7_O_4_NP)	4.03 × 10^5^
**57**	PE (18:2; 18:2-O1)	24.05	C_41_H_74_O_9_NP	754.5037		6.5	1.81				295.2275 (C_18_H_31_O_3_); 279.2326 (C_18_H_31_O_2_); 140.0108 (C_2_H_7_O_4_NP)	6.23 × 10^5^
**58**	PI (18:0; 18:2-O2)	24.23	C_45_H_83_O_15_P	893.5403		5.5	1.28				297.0380 (C_9_H_14_O_9_P); 293.2120 (C_18_H_29_O_3_); 283.2639 (C_18_H_35_O_2_); 241.0114 (C_6_H_10_O_8_P); 152.9949 (C_3_H_6_O_5_P)	8.33 × 10^5^
**61**	PE (16:0; 18:1-O2)	24.40	C_39_H_76_O_10_NP	748.5137		3.5	1.90				295.2276 (C_18_H_31_O_3_); 255.2328 (C_16_H_31_O_2_)	2.90 × 10^6^
**63**	PI (18:2; 14:0)	24.73	C_41_H_75_O_13_P	805.4873		5.5	0.74				577.2776 (C_27_H_46_O_11_P); 525.2458 (C_23_H_42_O_11_P); 363.1934 (C_17_H_32_O_6_P); 279.2328 (C_18_H_31_O_2_); 227.2009 (C_14_H_27_O_2_); 152.9946 (C_3_H_6_O_5_P)	1.27 × 10^6^
**64**	PC (18:2; 18:2-O2)	24.96	C_44_H_80_O_10_NP		858.5500	6.5	0.41				504.3091(C_25_H_47_O_7_NP); 415.2249 (C_21_H_36_O_6_P); 293.2121 (C_18_H_29_O_3_); 279.2328 (C_18_H_31_O_2_); 224.0685 (C_7_H_15_O_5_NP); 168.0422 (C_4_H_11_O_4_NP); 78.9578 (O_3_P)	5.96 × 10^6^
								814.5576	5.5	−2.76	184.0739 (C_5_H_15_O_4_NP); 86.0969 (C_5_H_12_N)	9.71 × 10^6^
**65**	PI (16:0; 18:3)	25.13	C_43_H_77_O_13_P	831.5034		6.5	1.21				575.2617 (C_27_H_44_O_11_P); 571.2906 (C_25_H_48_O_12_P); 551.2638 (C_25_H_46_O_11_P); 391.2257 (C_19_H_36_O_6_P); 297.0381 (C_9_H_14_O_9_P); 277.2172 (C_18_H_29_O_2_); 255.2327 (C_16_H_31_O_2_); 241.0116 (C_6_H_10_O_8_P); 223.0006 (C_6_H_8_O_7_P); 152.9947 (C_3_H_6_O_5_P)	1.98 × 10^6^
**67**	PI (18:2; 18:2)	25.69	C_45_H_79_O_13_P	857.5186		7.5	0.35				577.2777 (C_27_H_46_O_11_P); 415.2253 (C_21_H_36_O_6_P); 279.2326 (C_18_H_31_O_2_); 241.0116 (C_6_H_10_O_8_P); 152.9947 (C_3_H_6_O_5_P)	3.58 × 10^7^
**68**	PC (16:0; 18:2-O2)	26.20	C_42_H_80_O_10_NP		834.5507	4.5	1.29				480.3102 (C_23_H_47_O_7_NP); 293.2121 (C_18_H_29_O_3_); 255.2325 (C_16_H_31_O_2_); 224.0688 (C_7_H_15_O_5_NP); 168.0420 (C_4_H_11_O_4_NP); 78.9577 (O_3_P)	3.89 × 10^6^
								790.5574	3.5	−3.00	184.0735 (C_5_H_15_O_4_NP)	1.05 × 10^7^
**69**	PC (16:1; 18:2-O1)	26.28	C_42_H_78_O_9_NP		816.5399	5.5	1.63				295.2281 (C_18_H_31_O_3_); 253.2168 (C_16_H_29_O_2_)	8.92 × 10^4^
								772.5484	4.5	−1.09	184.0735 (C_5_H_15_O_4_NP); 86.0969 (C_5_H_12_N)	5.27 × 10^5^
**70**	PC (16:0; 18:3-O1)	26.35	C_42_H_78_O_9_NP		816.5410	5.5	2.97				293.2124 (C_18_H_29_O_3_); 255.2331 (C_16_H_31_O_2_)	1.08 × 10^5^
								772.5483	4.5	−0.54	184.0736 (C_5_H_15_O_4_NP); 86.0969 (C_5_H_12_N)	8.83 × 10^5^
**71**	PI (16:0; 18:2)	26.36	C_43_H_79_O_13_P	833.5178		5.5	−0.29				553.2784 (C_25_H_46_O_11_P); 391.2249 (C_19_H_36_O_6_P); 297.0373 (C_9_H_14_O_9_P); 279.2326 (C_18_H_31_O_2_); 255.2327 (C_16_H_31_O_2_); 223.0007 (C_6_H_8_O_7_P); 152.9948 (C_3_H_6_O_5_P)	1.93 × 10^8^
**72**	PE (18:2; 18:3)	26.46	C_41_H_72_O_8_NP	736.4924		7.5	1.61				279.2329 (C_18_H_31_O_2_); 277.2173 (C_18_H_29_O_2_); 196.0377 (C_5_H_11_O_5_NP)	9.95 × 10^5^
								738.5074	6.5	0.01	597.4871 (C_39_H_65_O_4_); 317.2488 (C_21_H_33_O_2_); 263.2383 (C_18_H_31_O)	5.05 × 10^6^
**73**	PC (18:2; 18:2-O1)	26.62	C_44_H_80_O_9_NP		842.5540	6.5	−0.18				295.2272 (C_18_H_31_O_3_); 279.2324 (C_18_H_31_O_2_); 224.0684 (C_7_H_15_O_5_NP); 168.0421 (C_4_H_11_O_4_NP); 78.9575 (O_3_P)	7.99 × 10^6^
								798.5632	5.5	−2.15	184.0735 (C_5_H_15_O_4_NP)	2.53 × 10^7^
**74**	PE (16:1; 18:2)	26.85	C_39_H_72_O_8_NP					714.5069	4.5	−0.67	573.4876 (C_37_H_65_O_4_); 263.2372 (C_18_H_31_O); 237.2217 (C_16_H_29_O)	1.34 × 10^6^
**76**	PE (15:0; 18:2)	27.07	C_38_H_72_O_8_NP					702.5070	3.5	0.27	561.4874 (C_36_H_65_O_4_); 337.2736 (C_21_H_37_O_3_); 263.2366 (C_18_H_31_O); 225.2212 (C_15_H_29_O)	1.89 × 10^6^
**77**	PE (16:0; 18:3)	27.25	C_39_H_72_O_8_NP	712.4924		5.5	1.67				277.2170 (C_18_H_29_O_2_); 255.2325 (C_16_H_31_O_2_); 196.0372 (C_5_H_11_O_5_NP)	2.28 × 10^6^
								714.5079	4.5	0.68	573.4866 (C_37_H_65_O_4_); 317.2495 (C_21_H_33_O_2_); 261.2214 (C_18_H_29_O)	1.53 × 10^6^
**78**	PI (18:1; 18:2)	27.38	C_45_H_81_O_13_P	859.5331		6.5	−0.59				579.2944 (C_27_H_48_O_11_P); 417.2408 (C_21_H_38_O_6_P); 297.0378 (C_9_H_14_O_9_P); 281.2481 (C_18_H_33_O_2_); 279.2328 (C_18_H_31_O_2_); 223.0007 (C_6_H_8_O_7_P); 152.9948 (C_3_H_6_O_5_P)	2.18 × 10^7^
**80**	PE (18:2; 18:2)	27.51	C_41_H_74_O_8_NP	738.5074		6.5	0.01				476.2777 (C_23_H_43_O_7_NP); 279.2325 (C_18_H_31_O_2_); 140.0105 (C_2_H_7_O_4_NP)	8.22 × 10^7^
								740.5222	5.5	−1.09	599.5033 (C_39_H_67_O_4_); 337.2737 (C_21_H_37_O_3_); 263.2367 (C_18_H_31_O)	1.39 × 10^8^
**82**	PI (16:0; 18:1)	28.21	C_43_H_81_O_13_P	835.5336		4.5	−0.10				553.2777 (C_27_H_46_O_11_P); 417.2412 (C_21_H_38_O_6_P); 391.2248 (C_19_H_36_O_6_P); 281.2480 (C_18_H_33_O_2_); 255.2324 (C_16_H_31_O_2_); 241.0113 (C_6_H_10_O_8_P); 223.0006 (C_6_H_8_O_7_P); 152.9948 (C_3_H_6_O_5_P)	1.21 × 10^7^
**84**	PC (18:1; 18:2-O1)	28.29	C_44_H_82_O_9_NP		844.5709	5.5	1.23				295.2275 (C_18_H_31_O_3_); 281.2482 (C_18_H_33_O_2_); 224.0689 (C_7_H_15_O_5_NP); 168.0420 (C_4_H_11_O_4_NP); 78.9575 (O_3_P)	2.75 × 10^6^
								800.5789	4.5	−1.34	184.0734 (C_5_H_15_O_4_NP)	1.03 × 10^7^
**85**	PE (16:0; 18:2)	28.32	C_39_H_74_O_8_NP	714.5071		4.5	−0.41				452.2778 (C_21_H_43_O_7_NP); 279.2325 (C_18_H_31_O_2_); 255.2325 (C_16_H_31_O_2_); 140.0105 (C_2_H_7_O_4_NP)	1.38 × 10^8^
								716.5218	3.5	−1.73	575.5035 (C_37_H_67_O_4_); 337.2744 (C_21_H_37_O_3_); 313.2735 (C_19_H_37_O_3_); 263.2371 (C_18_H_31_O); 239.2366 (C_16_H_31_O)	1.84 × 10^8^
**88**	PC (18:2; 14:0)	28.85	C_40_H_76_O_8_NP		774.5295	4.5	1.96				279.2326 (C_18_H_31_O_2_); 227.2009 (C_14_H_27_O_2_); 224.0691 (C_7_H_15_O_5_NP); 168.0421 (C_4_H_11_O_4_NP); 78.9577 (O_3_P)	2.66 × 10^6^
								730.5367	3.5	−1.99	184.0733 (C_5_H_15_O_4_NP)	1.28 × 10^7^
**89**	PI (18:0; 18:2)	28.88	C_45_H_83_O_13_P	861.5496		5.5	0.36				581.3091(C_27_H_50_O_11_P); 419.2560 (C_21_H_40_OP); 283.2640 (C_18_H_35_O_2_); 279.2324 (C_18_H_31_O_2_); 241.0113 (C_6_H_10_O_8_P); 223.0006 (C_6_H_8_O_7_P); 152.9947 (C_3_H_6_O_5_P)	6.53 × 10^7^
**90**	PC (18:2; 18:3)	28.97	C_44_H_78_O_8_NP		824.5453	7.5	2.04				279.2325 (C_18_H_31_O_2_); 277.2169 (C_18_H_29_O_2_); 224.0689 (C_7_H_15_O_5_NP); 168.0420 (C_4_H_11_O_4_NP); 78.9575 (O_3_P)	2.11 × 10^6^
								780.5530	6.5	−1.03	184.0734 (C_5_H_15_O_4_NP)	8.48 × 10^6^
**92**	PE (18:1; 18:2)	29.18	C_41_H_76_O_8_NP	740.5235		5.5	0.63				478.2933 (C_23_H_45_O_7_NP); 476.2767 (C_23_H_43_O_7_NP); 281.2482 (C_18_H_33_O_2_); 279.2327 (C_18_H_31_O_2_); 140.0107 (C_2_H_7_O_4_NP)	2.52 × 10^7^
								742.5374	4.5	−1.79	601.5190 (C_39_H_69_O_4_); 339.2884 (C_21_H_39_O_3_); 337.2736 (C_21_H_37_O_3_); 265.2527 (C_18_H_33_O); 263.2372 (C_18_H_31_O)	3.97 × 10^7^
**94**	PC (16:1; 18:2)	29.51	C_42_H_78_O_8_NP		800.5458	5.5	2.02				478.2954 (C_23_H_45_O_7_NP); 279.2325 (C_18_H_31_O_2_); 253.2168 (C_16_H_29_O_2_); 168.0418 (C_4_H_11_O_4_NP); 78.9575 (O_3_P)	7.10 × 10^6^
								756.5530	4.5	−1.00	184.0734 (C_5_H_15_O_4_NP)	1.70 × 10^7^
**95**	PC (18:2; 18:2)	30.03	C_44_H_80_O_8_NP		826.5592	6.5	−0.73				504.3087 (C_25_H_47_O_7_NP); 279.2325 (C_18_H_31_O_2_); 224.0687 (C_7_H_15_O_5_NP); 168.0422 (C_4_H_11_O_4_NP); 78.9575 (O_3_P)	1.57 × 10^8^
								782.5679	5.5	−2.38	184.0734 (C_5_H_15_O_4_NP); 124.9998 (C_2_H_6_O_4_P); 86.0969 (C_5_H_12_N)	4.45 × 10^8^
**97**	PC (16:0; 18:2)	30.71	C_42_H_80_O_8_NP		802.5586	4.5	0.00				480.3074 (C_23_H_47_O_7_NP); 279.2326 (C_18_H_31_O_2_); 255.2326 (C_16_H_31_O_2_); 168.0423 (C_4_H_11_O_4_NP); 78.9574 (O_3_P)	1.46 × 10^8^
								758.5672	3.5	−2.94	184.0732 (C_5_H_15_O_4_NP); 124.9998(C_2_H_6_O_4_P); 86.0969 (C_5_H_12_N)	4.71 × 10^8^
**100**	PC (18:2; 18:1)	31.41	C_44_H_82_O_8_NP		828.5751	5.5	−0.46				506.3248 (C_25_H_49_O_7_NP); 281.2485 (C_18_H_33_O_2_); 279.2326 (C_18_H_31_O_2_); 224.0683 (C_7_H_15_O_5_NP); 168.0421 (C_4_H_11_O_4_NP); 78.9576 (O_3_P)	8.08 × 10^7^
								784.5834	4.5	−2.09	184.0734 (C_5_H_15_O_4_NP); 124.9999 (C_2_H_6_O_4_P); 86.0969 (C_5_H_12_N)	3.21 × 10^8^
**103**	PC (16:0; 18:1)	32.50	C_42_H_82_O_8_NP		804.5753	3.5	0.27				480.3090 (C_23_H_47_O_7_NP); 281.2482 (C_18_H_33_O_2_); 255.2327(C_16_H_31_O_2_); 168.0418 (C_4_H_11_O_4_NP); 78.9576 (O_3_P)	4.20 × 10^7^
								760.5839	2.5	−1.53	184.0734 (C_5_H_15_O_4_NP); 125.0000 (C_2_H_6_O_4_P); 86.0969 (C_5_H_12_N)	1.27 × 10^8^
**104**	PC (18:1; 18:1)	33.09	C_44_H_84_O_8_NP		830.5915	4.5	0.45				506.3229 (C_25_H_49_O_7_NP); 281.2484 (C_18_H_33_O_2_); 224.0690 (C_7_H_15_O_5_NP); 168.0423 (C_4_H_11_O_4_NP); 78.0575 (O_3_P)	5.15 × 10^7^
								786.5992	3.5	−1.97	184.0733 (C_5_H_15_O_4_NP); 124.9998 (C_2_H_6_O_4_P); 86.0969 (C_5_H_12_N)	1.94 × 10^8^
**107**	PEth (18:2; 18:2)	34.55	C_41_H_73_O_8_P	723.4984		6.5	3.34				461.2671 (C_23_H_42_O_7_P); 279.2325 (C_18_H_31_O_2_); 181.0261 (C_5_H_10_O_5_P); 152.9948 (C_3_H_6_O_5_P); 124.9998 (C_2_H_6_O_4_P)	3.21 × 10^6^
**109**	PC (18:0; 18:1)	35.05	C_44_H_86_O_8_NP		832.6086	3.5	2.18				508.3392 (C_25_H_51_O_7_NP); 283.2638 (C_18_H_35_O_2_); 281.2484 (C_18_H_33_O_2_); 224.0689 (C_7_H_15_O_5_NP); 168.0419 (C_4_H_11_O_4_NP); 78.9577 (O_3_P)	3.00 × 10^6^
								788.6147	2.5	−2.14	184.0733 (C_5_H_15_O_4_NP); 124.9999 (C_2_H_6_O_4_P); 86.0969 (C_5_H_12_N)	7.85 × 10^7^
**111**	PEth (16:0; 18:2)	36.04	C_39_H_73_O_8_P	699.4982		4.5	3.20				437.2660 (C_21_H_42_O_7_P); 279.2325 (C_18_H_31_O_2_); 255.2325 (C_16_H_31_O_2_); 181.0263 (C_5_H_10_O_5_P); 152.9947 (C_3_H_6_O_5_P); 124.9998 (C_2_H_6_O_4_P)	2.89 × 10^6^
**112**	PEth (16:0; 18:2)	36.12	C_41_H_74_O_8_P	725.5139		5.5	3.22				461.2687 (C_23_H_42_O_7_P); 279.2328 (C_18_H_31_O_2_); 281.2484 (C_18_H_33_O_2_); 181.0264 (C_5_H_10_O_5_P); 124.9996 (C_2_H_6_O_4_P)	9.34 × 10^5^
**113**	PC (20:0; 18:2)	36.49	C_46_H_88_O_8_NP		858.6234	4.5	1.80				311.2954 (C_20_H_39_O_2_); 279.2325 (C_18_H_31_O_2_); 224.0688 (C_7_H_15_O_5_NP); 168.0423 (C_4_H_11_O_4_NP); 78.9577 (O_3_P)	7.24 × 10^5^
								814.6314	3.5	−0.76	184.0734 (C_5_H_15_O_4_NP); 124.9999 (C_2_H_6_O_4_P); 86.0969 (C_5_H_12_N)	6.00 × 10^6^

Note: abbreviations: l-PCs, lyso-phosphatidylcholines; NA-GPEs, N-acylglycerophosphatidylethanolamines; l-PEs, lyso-phosphatidylethanolamines; l-PIs, lyso-phosphatidylinositols; l-PA, lyso-phosphatidic acid; PCs, phosphatidylcholines; PEs, phosphatidylethanolamines; PIs, phosphatidylinositols; PEths, phosphatidylethanols. The oxidized acyl chains are abbreviated as, e.g., 18:1-O1 and 18:1-O2 to indicate an 18-carbon chain length with one double bond equivalent and either one or two additional oxygen atoms beyond the carbonyl group. NL: normalized level.

**Table 2:** Sphingolipids tentatively identified in the EtOH extract of *C. pepo* var. *styriaca* seeds.

Sphingolipids												
N°	Compound	R_t_ (min)	Molecular Formula	[M–H]^−^	[(M + HCO_2_H)–H]^−^	RDB	ppm	[M + H]^+^	RDB	ppm	HRMS/MS	MS Peak Intensity (NL)
**5**	LCB (t18:1)	9.44	C_18_H_37_O_3_N					316.2848	0.5	0.82	298.2789 (C_18_H_36_O_2_N); 280.2626 (C_18_H_34_ON); 262.2529 (C_18_H_32_N); 74.0607 (C_3_H_8_ON); 60.0450 (C_2_H_6_ON)	8.18 × 10^5^
**9**	LCB (d18:0)	10.45	C_18_H_39_O_2_N					302.3056	−0.5	0.70	284.2954 (C_18_H_38_ON); 254.2852 (C_17_H_36_N); 106.0866 (C_4_H_12_O_2_N); 102.0920 (C_5_H_12_ON); 88.0762 (C_4_H_10_ON)	5.77 × 10^5^
**11**	LCB (t18:0)	10.83	C_18_H_39_O_3_N					318.3005	−0.5	0.84	300.2880 (C_18_H_38_O_2_N); 282.2791 (C_18_H_36_ON); 270.2794 (C_17_H_36_ON); 264.2690 (C_18_H_34_N); 122.0814 (C_4_H_12_O_3_N); 90.0556 (C_3_H_8_O_2_N); 74.0606 (C_3_H_8_ON); 60.0450 (C_2_H_6_ON)	1.45 × 10^6^
**16**	LCB (*N*-acetyl-d18:1)	12.01	C_20_H_39_O_3_N					342.3001	1.5	−0.47	298.2736 (C_18_H_36_O_2_N); 280.2636 (C_18_H_34_ON); 262.2528 (C_18_H_32_N); 240.2324 (C_15_H_30_ON); 88.0762 (C_4_H_10_ON); 60.0450 (C_2_H_6_ON)	3.68 × 10^5^
**18**	LCB (t19:1)	12.36	C_19_H_39_O_3_N					330.2998	0.5	−1.03	312.28973 (C_19_H_38_O_2_N); 300.2903 (C_18_H_38_O_2_N); 294.2792 (C_19_H_36_ON); 282.2784 (C_18_H_36_ON); 270.2806 (C_17_H_36_ON); 264.2697 (C_18_H_34_N); 74.0606 (C_3_H_8_ON); 60.0450 (C_2_H_6_ON)	5.34 × 10^5^
**62**	HexCer (d18:2; h16:0)	24.51	C_40_H_75_O_9_N	712.5366	758.5425	4.5	1.14				550.4831 (C_34_H_64_O_4_N); 532.4731 (C_34_H_62_O_3_N); 296.2590 (C_18_H_34_O_2_N); 271.2272 (C_16_H_31_O_3_); 270.2439 (C_16_H_32_O_2_N); 253.2172 (C_16_H_29_O_2_); 225.2219 (C_15_H_29_O)	1.95 × 10^7^
								714.5497	3.5	−2.41	534.4886 (C_34_H_64_O_3_N); 516.4769 (C_34_H_62_O_2_N); 272.2586 (C_16_H_34_O_2_N); 262.2530 (C_18_H_32_N)	8.73 × 10^6^
**66**	HexCer (d18:2; N16:0)	25.62	C_40_H_75_O_8_N	696.5406	742.5482	4.5	−0.44				534.4885 (C_34_H_64_O_3_N); 296.2598 (C_18_H_34_O_2_N); 255.2329 (C_16_H_31_O_2_); 254.2488 (C_16_H_32_ON); 237.2213 (C_16_H_29_O); 235.2061 (C_16_H_27_O)	1.27 × 10^6^
								698.5560	3.5	−0.74	518.44988 (C_34_H_64_O_2_N); 280.2632 (C_18_H_34_ON); 262.2529 (C_18_H_32_N)	1.00 × 10^6^
**81**	Cer (t18:1; N18:2)	27.71	C_36_H_67_O_4_N	576.4986	622.5057	4.5	−0.06				334.2759 (C_21_H_36_O_2_N); 322.2744 (C_20_H_36_O_2_N); 320.2588 (C_20_H_34_O_2_N); 279.2327 (C_18_H_31_O_2_); 278.2493 (C_18_H_32_ON); 265.2162 (C_17_H_29_O_2_)	1.54 × 10^5^
**83**	Cer (t18:1; N16:0)	28.27	C_34_H_67_O_4_N	552.4990	598.5052	2.5	0.64				516.4817 (C_34_H_62_O_2_N); 310.2748 (C_19_H_36_O_2_N); 298.2749 (C_18_H_36_O_2_N); 265.2169 (C_17_H_29_O2); 255.2327 (C_16_H_31_O_2_); 254.2492 (C_16_H_32_ON); 253.2189 (C_16_H_29_O_2_)	3.63 × 10^5^
								554.5148	1.5	0.98	536.5062 (C_34_H_66_O_3_N); 518.4940 (C_34_H_64_O_2_N); 316.2837 (C_18_H_38_O_3_N); 298.2744 (C_18_H_36_O_2_N); 280.2630 (C_18_H_34_ON); 262.2535 (C_18_H_32_N); 250.2528 (C_17_H_32_N); 60.0451 (C_2_H_6_ON)	4.46 × 10^5^
**86**	Cer (d18:2; h16:0)	28.41	C_34_H_65_O_4_N	550.4834	596.4892	3.5	0.75				312.2528 (C_18_H_34_O_3_N); 296.2593 (C_18_H_34_O_2_N); 278.2489 (C_18_H_32_ON); 271.2280 (C_16_H_31_O_3_); 270.2436 (C_16_H_32_O_2_N); 253.2189 (C_16_H_29_O_2_); 225.2224 (C_15_H_29_O)	3.14 × 10^5^
**91**	Cer (d18:0; h16:0)	29.02	C_34_H_69_O_4_N	554.5154	600.5209	1.5	2.08				310.2751 (C_19_H_36_O_2_N); 298.2751 (C_18_H_36_O_2_N); 267.2332 (C_17_H_31_O_2_); 255.2327 (C_16_H_31_O_2_)	2.35 × 10^5^
								556.5301	0.5	0.38	538.5208 (C_34_H_68_O_3_N); 318.2993 (C_18_H_40_O_3_N); 300.2895 (C_18_H_38_O_2_N); 282.2791 (C_18_H_36_ON); 264.2684 (C_18_H_34_N); 256.2640 (C_16_H_34_ON); 60.0451 (C_2_H_6_ON)	4.04 × 10^5^
**96**	Cer (d18:2; N16:0)	30.30	C_34_H_65_O_3_N	534.4883	580.4941	3.5	0.50				296.2590 (C_18_H_34_O_2_N); 280.2647 (C_18_H_34_ON); 261.2222 (C_18_H_29_O); 255.2326 (C_16_H_31_O_2_); 254.2488 (C_16_H_32_ON); 237.2210 (C_16_H_29_O); 235.2065 (C_16_H_27_O)	1.12 × 10^6^
								536.5034	2.5	−0.67	280.2640 (C_18_H_34_ON); 262.2529 (C_18_H_32_N)	4.84 × 10^5^
**101**	Cer (d18:0; N16:0)	31.68	C_34_H_69_O_3_N	538.5195	584.5258	1.5	0.29				280.2641 (C_18_H_34_ON); 255.2335 (C_16_H_31_O_2_)	7.33 × 10^5^
								540.5350	0.5	0.02	522.5254 (C_34_H_68_O_2_N); 504.5153 (C_34_H_66_ON); 302.3037 (C_18_H_40_O_2_N); 284.29503 (C_18_H_38_ON); 266.2843 (C_18_H_36_N); 256.2637 (C_16_H_34_ON); 60.0451 (C_2_H_6_ON)	1.16 × 10^6^
**117**	Cer-(EO)LCB (t18:1/39:5) (t18:1; O21:2; E18:3)	39.06	C_57_H_101_O_7_N					912.7623	7.5	−3.61	597.4878 (C_39_H_65_O_4_); 337.2746 (C_21_H_37_O_3_); 261.2213 (C_18_H_29_O); 243.2106 (C_18_H_27_)	8.81 × 10^7^
**118**	Cer-(EO) LCB (t18:3/39:4) (t18:3; O21:2; E18:2)	39.40	C_57_H_99_O_7_N					910.7482	8.5	−1.99	599.5040 (C_39_H_67_O_4_); 337.2736 (C_21_H_37_O_3_); 277.2161 (C_18_H_29_O_2_)	4.19 × 10^7^
**120**	Cer-(EO) LCB (t18:1/39:4) (t18:1; O21:2; E18:2)	39.45	C_57_H_103_O_7_N					914.7795	6.5	−1.97	599.5033 (C_39_H_67_O_4_); 337.2753 (C_21_H_37_O_3_); 277.2149 (C_18_H_29_O_2_); 261.2215 (C_18_H_29_O)	7.45 × 10^7^
**122**	Cer-(EO) LCB (t18:2/37:2) (t18:2; O19:0; E18:2)	39.64	C_55_H_101_O_7_N					888.7641	5.5	−1.66	575.5041 (C_37_H_67_O_4_); 313.2748 (C_19_H_37_O_3_); 261.2214 (C_18_H_29_O); 243.2113 (C_18_H_27_)	4.30 × 10^7^
**126**	Cer-(EO) LCB (t18:3/39:3) (t18:3; O21:2; E18:1)	40.13	C_57_H_101_O_7_N					912.7633	7.5	−2.49	601.5208 (C_39_H_69_O_4_); 337.2743 (C_21_H_37_O_3_); 277.2162 (C_18_H_29_O_2_)	6.41 × 10^7^
**129**	Cer-(EO) LCB (t18:1/39:3) (t18:1; O21:1; E18:2)	40.25	C_57_H_105_O_7_N					916.7944	5.5	−2.79	601.5192 (C_39_H_69_O_4_); 339.2890 (C_21_H_39_O_3_)	5.66 × 10^7^
**131**	Cer-(EO) LCB (t18:2/37:2) (t18:2; O21:2; E16:0)	40.70	C_55_H_101_O_7_N					888.7639	5.5	−1.92	575.5037 (C_37_H_67_O_4_); 337.2727 (C_21_H_37_O_3_)	3.54 × 10^7^
**132**	Cer-(EO) LCB (d16:1/33:2) (d16:1; O17:1; E16:1)	40.73	C_49_H_91_O_6_N					790.6922	4.5	−0.31	519.4400 (C_33_H_59_O_4_); 283.2270 (C_17_H_31_O_3_); 237.2212 (C_16_H_29_O); 219.2111 (C_16_H_27_)	7.52 × 10^5^
**133**	Cer-(EO) LCB (t18:2/39:3) (t18:2; O21:2; E18:1)	40.83	C_57_H_103_O_7_N					914.7786	6.5	−2.97	601.5177 (C_39_H_69_O_4_); 337.2744 (C_21_H_37_O_3_); 277.2161 (C_18_H_29_O_2_)	5.59 × 10^7^
**137**	Cer-(EO) LCB (d16:1/32:1) (d16:1/O16:0; E16:1)	41.09	C_48_H_91_O_6_N					778.6933	3.5	1.07	507.4410 (C_32_H_59_O_4_); 271.2276 (C_16_H_31_O_3_); 237.2216 (C_16_H_29_O); 219.2115 (C_16_H_27_)	1.30 × 10^6^
**138**	Cer-(EO) LCB (t18:1/39:2) (t18:1/O21:1; E18:1)	41.12	C_57_H_107_O_7_N					918.8104	4.5	−2.35	603.5341 (C_39_H_71_O_4_); 339.2892 (C_21_H_39_O_3_); 269.2373 (C_18_H_31_O); 245.2263 (C_18_H_29_)	4.20 × 10^7^
**139**	Cer-(EO) LCB (d18:1/31:1) (d18:1/O16:0; E15:1)	41.22	C_49_H_93_O_6_N					792.7083	3.5	0.25	493.4266 (C_31_H_57_O_4_); 271.2267 (C_16_H_31_O_3_); 237.2213 (C_16_H_29_O); 219.2110 (C_16_H_27_)	7.92 × 10^6^
**140**	Cer-(EO) LCB (d16:1/34:2) (d16:1; O18:1; E16:1)	41.23	C_50_H_93_O_6_N					804.7079	4.5	−0.20	533.4571 (C_34_H_61_O_4_); 297.2426 (C_18_H_33_O_3_); 237.2211 (C_16_H_29_O); 219.2110 (C_16_H_27_)	1.35 × 10^6^
**141**	Cer-(EO) LCB (d16:1/31:0) (d16:1; O15:0; E16:0)	41.28	C_47_H_91_O_6_N					766.6924	2.5	0.68	495.4406 (C_31_H_59_O_4_); 257.2116 (C_15_H_29_O_3_); 239.2376 (C_16_H_31_O); 211.2059 (C_14_H_27_O); 183.1748 (C_12_H_23_O)	4.19 × 10^6^
**142**	Cer-(EO) LCB (d18:2/33:1) (d18:2; O17:0; E16:1)	41.28	C_51_H_95_O_6_N					818.7226	4.5	−1.42	521.4575 (C_33_H_61_O_4_); 285.2422 (C_17_H_33_O_3_)	1.40 × 10^6^
**143**	Cer-(EO) LCB (d18:2/35:2) (d18:2; O17:0; E18:2)	41.39	C_53_H_97_O_6_N					844.7396	5.5	0.24	547.4724 (C_35_H_63_O_4_); 285.2430 (C_17_H_33_O_3_); 263.2374 (C_18_H_31_O)	1.44 × 10^7^
**144**	Cer-(EO) LCB (d16:1/33:1) (d16:1; O17:0; E16:1)	41.39	C_49_H_93_O_6_N					792.7079	3.5	−0.21	521.4574 (C_33_H_61_O_4_); 285.2430 (C_17_H_33_O_3_); 237.2218 (C_16_H_29_O); 219.2105 (C_16_H_27_)	7.92 × 10^6^
**145**	Cer-(EO) LCB (t18:2/39:2) (t18:2; O21:1; E18:1)	41.40	C_57_H_105_O_7_N					916.7953	5.5	−1.72	603.5350 (C_39_H_71_O_4_); 339.2905 (C_21_H_39_O_3_); 279.2322 (C_18_H_31_O_2_); 261.2214 (C_18_H_29_O)	4.82 × 10^7^
**146**	Cer-(EO) LCB (d16:1/33:2) (d16:1; O17:1; E16:1)	41.43	C_51_H_95_O_6_N					818.7231	4.5	−0.84	519.4418 (C_33_H_59_O_4_); 283.2271 (C_17_H_31_O_3_); 237.2213 (C_16_H_29_O);	3.86 × 10^6^
**147**	Cer-(EO) LCB (d16:1/35:2) (d16:1; O19:1; E16:1)	41.54	C_51_H_95_O_6_N					818.7230	4.5	−0.98	547.4715 (C_35_H_63_O_4_); 311.2584 (C_19_H_35_O_3_); 237.2221 (C_16_H_29_O)	2.89 × 10^6^
**148**	Cer-(EO) LCB (d16:1/39:4) (d16:1; O21:2; E18:2)	41.60	C_57_H_101_O_6_N					896.7688	7.5	−1.52	599.5032 (C_39_H_67_O_4_); 337.2740 (C_21_H_37_O_3_); 319.2634 (C_21_H_35_O_2_); 263.2372 (C_18_H_31_O); 245.2267 (C_18_H_29_)	3.02 × 10^8^
**149**	Cer-(EO) LCB (d16:1/34:1) (d16:1; O18:0; E16:1)	41.86	C_50_H_95_O_6_N					806.7240	3.5	0.29	535.4723 (C_34_H_63_O_4_); 299.2587 (C_18_H_35_O_3_); 237.2212 (C_16_H_29_O)	3.71 × 10^6^
**150**	Cer-(EO) LCB (t18:2/39:1) (t18:2; O21:1; E18:0)	41.91	C_57_H_107_O_7_N					918.8107	4.5	−2.01	605.5483 (C_39_H_73_O_4_); 339.2897 (C_21_H_39_O_3_); 265.2527 (C_18_H_33_O)	1.51 × 10^7^
**151**	Cer-(EO) LCB (d18:2/33:0) (d18:2; O17:0; E16:0)	41.96	C_51_H_97_O_6_N					820.7383	3.5	−0.64	523.4726 (C_33_H_63_O_4_); 285.5430 (C_17_H_33_O_3_); 239.2374 (C_16_H_31_O);	9.42 × 10^6^
**152**	Cer-(EO) LCB (d18:2/37:2) (d18:2; O19:0; E18:2)	41.98	C_55_H_101_O_6_N					872.7686	5.5	−1.73	575.5036 (C_37_H_67_O_4_); 313.2738 (C_19_H_37_O_3_); 263.2366 (C_18_H_31_O); 239.2373 (C_16_H_31_O)	2.86 × 10^8^
**153**	Cer-(EO) LCB (d18:2/39:3) (d18:2; O21:2; E18:1)	42.00	C_57_H_103_O_6_N					898.7841	6.5	−1.95	601.5194 (C_39_H_69_O_4_); 337.2735 (C_21_H_37_O_3_); 265.2527 (C_18_H_33_O)	2.39 × 10^8^
**154**	Cer-(EO) LCB (d16:1/35:1) (d16:1; O19:0; E16:1)	42.03	C_51_H_97_O_6_N					820.7385	3.5	−1.16	549.4878 (C_35_H_65_O_4_); 313.2736 (C_19_H_37_O_3_); 237.2214 (C_16_H_29_O)	9.42 × 10^6^
**155**	Cer-(EO) LCB (d16:0/39:4) (d16:0; O21:2; E18:2)	42.05	C_55_H_101_O_6_N					872.7688	5.5	−2.19	599.5030 (C_39_H_67_O_4_); 337.2743 (C_21_H_37_O_3_); 263.2368 (C_18_H_31_O)	2.75 × 10^8^

Note: abbreviations: LCBs, long chain bases; HexCers. Hexosylceramides; Cers, ceramides; Cer-(EO)LCB, Cer = ceramide, E = second fatty acid, esterified at the ω-position of the *N*-acylated fatty acid, O = α,ω-dihydroxylated fatty acid *N*-acylated to the LCB. The nomenclature for long-chain bases (LCBs) consists of an initial letter, ‘d’ for dihydroxylated bases (e.g., d18:0) or ‘t’ for trihydroxylated ones (e.g., t18:1), followed by two numbers separated by a colon to indicate the number of carbons and double bonds. Additionally, in Cer and HexCer nomenclature, *N*-acylated fatty chains are designated by adding the letter ‘h’ or ‘N’ to the carbon chain length and double bond equivalents to indicate whether they are hydroxylated, usually at the C-2 position (e.g., HexCer(d18:2; h16:0)), or non-hydroxylated, respectively (e.g., Cer(t18:1; N18:2)). In Cer-(EO)LCB nomenclature, the first pair of brackets contains the LCB type followed by a slash and two numbers separated by a colon, indicating the total carbon chain length and number of double bonds of the fatty acyl portion (e.g., d16:1/34:1). The second pair of brackets designates the esterified structure: the α,ω-dihydroxylated fatty acid *N*-acylated to the LCB is indicated by ‘O’, while the fatty acid esterified at the ω-position of ‘O’ is indicated by ‘E’, with both followed by their respective carbon chain length and double bonds (e.g., d16:1; O18:0; E16:1). NL: normalized level.

**Table 3:** Acylglicerols tentatively identified in the EtOH extract of *C. pepo* var. *styriaca* seeds.

Acylglicerols												
N°	Compound	R_t_ (min)	Molecular Formula	[M–H]^−^	[(M + HCO_2_H)–H]^−^	RDB	ppm	[M + H]^+^	RDB	ppm	HRMS/MS	MS Peak Intensity (NL)
**87**	MGE (O-18:2; 18:2)	28.55	C_39_H_70_O_4_					603.5341	4.5	−1.04	263.2372 (C_18_H_31_O); 267.2681 (C_18_H_35_O)	2.60 × 10^7^
**93**	MGE (O-16:2; 18:2)	29.41	C_37_H_66_O_4_					575.5032	4.5	−0.27	263.2368 (C_18_H_31_O); 239.2370 (C_16_H_31_O)	1.41 × 10^7^
**98**	DG (18:1; 18:3)	30.92	C_39_H_68_O_5_					617.5137	5.5	−0.36	599.5008 (C_39_H_67_O_4_); 339.2895 (C_21_H_39_O_3_); 335.2587 (C_21_H_35_O_3_); 265.2530 (C_18_H_33_O); 261.2217 (C_18_H_29_O)	1.22 × 10^7^
**102**	DG (16:0; 18:3)	32.06	C_37_H_66_O_5_					591.4973	4.5	−0.02	573.4902 (C_37_H_65_O_4_); 335.2585 (C_21_H_35_O_3_); 313.2738 (C_19_H_37_O_3_); 279.2320 (C_18_H_31_O_2_); 261.2212 (C_18_H_29_O); 239.2372 (C_16_H_31_O)	1.40 × 10^7^
**105**	DG (18:1; 18:3)	33.76	C_39_H_68_O_5_					617.5134	5.5	−0.84	599.5030 (C_39_H_67_O_4_); 339.2907 (C_21_H_39_O_3_); 335.2577 (C_21_H_35_O_3_); 261.2213 (C_18_H_29_O)	2.60 × 10^8^
**106**	DG (16:0; 18:2)	34.52	C_37_H_68_O_5_					593.5134	3.5	−0.88	575.5017 (C_37_H_67_O_4_); 337.2733 (C_21_H_37_O_3_); 313.2737 (C_19_H_37_O_3_); 263.2366 (C_18_H_31_O); 239.2364 (C_16_H_31_O)	1.33 × 10^7^
**108**	DG (18:1; 18:2)	34.78	C_39_H_70_O_5_					619.5289	4.5	−0.98	601.5196 (C_39_H_69_O_4_); 339.2893 (C_21_H_39_O_3_); 337.2737 (C_21_H_37_O_3_); 265.2519 (C_18_H_33_O); 263.2368 (C_18_H_31_O)	5.98 × 10^7^
**110**	DG (18:1; 18:1)	35.93	C_39_H_72_O_5_					621.5454	3.5	0.25	603.5329 (C_39_H_71_O_4_); 339.2893 (C_21_H_39_O_3_); 265.2521 (C_18_H_33_O)	6.32 × 10^6^
**114**	TG (14:0; 18:2; 18:3)	38.69	C_53_H_92_O_6_					825.6965	7.5	−0.17	615.5013 (C_39_H_67_O_5_); 597.4877 (C_39_H_65_O_4_); 317.2481 (C_21_H_33_O_2_); 285.2433 (C_17_H_33_O_3_); 261.2213 (C_18_H_29_O); 211.2062 (C_14_H_27_O)	5.18 × 10^6^
**115**	TG (16:1; 18:2; 18:3)	38.86	C_55_H_94_O_6_					851.7119	8.5	−0.48	597.4864 (C_39_H_65_O_4_); 573.4883 (C_37_H_65_O_4_); 571.4706 (C_37_H_63_O_4_); 337.2750 (C_21_H_37_O_3_); 317.2483 (C_21_H_33_O_2_); 311.2588 (C_19_H_35_O_3_); 263.2375 (C_18_H_31_O); 261.2209 (C_18_H_29_O); 237.2214 (C_16_H_29_O)	8.82 × 10^6^
**116**	TG (18:2; 18:2; 18:3)	39.05	C_57_H_96_O_6_					877.7266	9.5	−1.60	615.5004 (C_39_H_67_O_5_); 599.5040 (C_39_H_67_O_4_); 597.4880 (C_39_H_65_O_4_); 337.2738 (C_21_H_37_O_3_); 317.2489 (C_21_H_33_O_2_); 263.2370 (C_18_H_31_O); 261.2219 (C_18_H_29_O)	2.95 × 10^8^
**119**	TG (18:2; 18:2; 18:3-O1)	39.44	C_57_H_96_O_7_					893.7214	9.5	−1.89	613.4834 (C_39_H_65_O_5_); 599.5052 (C_39_H_67_O_4_); 337.2729 (C_21_H_37_O_3_); 277.2164 (C_18_H_29_O_2_); 263.2367 (C_18_H_31_O)	1.84 × 10^8^
**121**	TG (16:0; 18:2; 18:3)	39.62	C_55_H_96_O_6_					853.7266	7.5	−1.58	615.4980 (C_39_H_67_O_5_); 597.4879 (C_39_H_65_O_4_); 317.2478 (C_21_H_33_O_2_); 313.2747 (C_19_H_37_O_3_); 263.2368 (C_18_H_31_O); 261.2215 (C_18_H_29_O); 239.2371 (C_16_H_31_O)	1.94 × 10^8^
**123**	TG (18:1; 18:2; 18:3)	39.78	C_57_H_98_O_6_					879.7416	8.5	−2.32	601.5181 (C_39_H_69_O_4_); 599.5039 (C_39_H_67_O_4_); 597.4873 (C_39_H_65_O_4_); 339.2897 (C_21_H_39_O_3_); 337.2738 (C_21_H_37_O_3_); 265.2530 (C_18_H_33_O); 263.2373 (C_18_H_31_O); 261.2218 (C_18_H_29_O)	2.47 × 10^8^
**124**	TG (16:0; 18:2; 18:3-O1)	40.01	C_55_H_96_O_7_					869.7217	7.5	−1.38	613.4841 (C_39_H_65_O_5_); 589.4836 (C_37_H_65_O_5_); 575.5016 (C_37_H_67_O_4_); 351.2537 (C_21_H_35_O_4_); 337.2749 (C_21_H_37_O_3_); 313.2727 (C_19_H_37_O_3_); 295.2271 (C_18_H_31_O_3_); 277.2165 (C_18_H_29_O_2_); 263.2373 (C_18_H_31_O); 239.2371 (C_16_H_31_O)	1.54 × 10^8^
**125**	TG (16:0; 18:1; 18:3)	40.13	C_55_H_98_O_6_					855.7429	6.5	−0.88	599.5025 (C_39_H_67_O_4_); 577.5186 (C_37_H_69_O_4_); 573.4921 (C_37_H_65_O_4_); 339.2911 (C_21_H_39_O_3_); 335.2609 (C_21_H_35_O_3_); 317.2482 (C_21_H_33_O_2_); 313.2736 (C_19_H_37_O_3_); 265.2533 (C_18_H_33_O); 261.2212 (C_18_H_29_O)	5.47 × 10^7^
**127**	TG (18:1; 18:2; 18:3-O1)	40.15	C_57_H_98_O_7_					895.7369	8.5	−1.78	615.4997 (C_39_H_67_O_5_); 613.4845 (C_39_H_65_O_5_); 601.5207 (C_39_H_69_O_4_); 351.2531 (C_21_H_35_O_4_); 339.2895 (C_21_H_39_O_3_); 337.2745 (C_21_H_37_O_3_); 277.2163 (C_18_H_29_O_2_); 265.2516 (C_18_H_33_O); 263.2378 (C_18_H_31_O)	1.90 × 10^8^
**128**	TG (18:1; 18:1; 18:3)	40.21	C_57_H_100_O_6_					881.7575	7.5	−1.98	603.5333 (C_39_H_71_O_4_); 599.5027 (C_39_H_67_O_4_); 339.2898 (C_21_H_39_O_3_); 265.2523 (C_18_H_33_O); 261.2220 (C_18_H_29_O)	8.84 × 10^7^
**130**	TG (18:0; 18:2; 18:3)	40.58	C_57_H_100_O_6_					881.7576	7.5	−1.92	603.5333 (C_39_H_71_O_4_); 597.4877 (C_39_H_65_O_4_); 341.3072 (C_21_H_41_O_3_); 337.2743 (C_21_H_37_O_3_); 263.2366 (C_18_H_31_O) 261.2215 (C_18_H_29_O)	1.14 × 10^8^
**134**	TG (18:1; 18:1; 18:3-O1)	40.88	C_57_H_100_O_7_					897.7524	7.5	−1.96	615.4972 (C_39_H_67_O_5_); 603.5349 (C_39_H_71_O_4_); 351.2534 (C_21_H_35_O_4_); 339.2890 (C_21_H_39_O_3_); 277.2161 (C_18_H_29_O_2_); 265.2524 (C_18_H_33_O)	7.77 × 10^7^
**135**	TG (16:0; 18:0; 18:3)	40.98	C_55_H_100_O_6_					857.7595	5.5	−0.41	601.5190 (C_39_H_69_O_4_); 579.5336 (C_37_H_71_O_4_); 573.4866 (C_37_H_65_O_4_); 341.3055 (C_21_H_41_O_3_); 317.2481 (C_21_H_33_O_2_); 313.2744 (C_19_H_37_O_3_); 261.2213 (C_18_H_29_O); 239.2375 (C_16_H_31_O);	4.37 × 10^6^
**136**	TG (18:0; 18:1; 18:3)	41.08	C_57_H_102_O_6_					883.7739	6.5	−1.12	605.5509 (C_39_H_73_O_4_); 601.5182 (C_39_H_69_O_4_); 599.5053 (C_39_H_67_O_4_); 341.3061 (C_21_H_41_O_3_); 339.2905 (C_21_H_39_O_3_); 265.2526 (C_18_H_33_O); 261.2215 (C_18_H_29_O)	2.83 × 10^7^

Note: abbreviations: MGEs, monoacylglycerol ethers; DGs, diacylglycerols; TGs, triacylglycerols. In MGEs, alkyl chains are abbreviated as, e.g., O-18:2 to indicate an 18-carbon chain length with two double bond equivalents, ether-linked to one glycerol oxygen. In TGs, oxidized acyl chains are abbreviated as 18:3-O1 to indicate an 18-carbon chain length with three double bond equivalents and one additional oxygen atom beyond the carbonyl group. NL: normalized level.

**Table 4:** Triterpenoids tentatively identified in the EtOH extract of *C. pepo* var. *styriaca* seeds.

Triterpenoids												
N°	Compound	R_t_ (min)	Molecular Formula	[M–H]^−^	[(M + HCO_2_H)–H]^−^	RDB	ppm	[M + H]^+^	RDB	ppm	HRMS/MS	MS Peak Intensity (NL)
**36**	*O*-aminobenzoyl-multifloren-triol	17.10	C_37_H_55_O_4_N					578.4201	10.5	0.36	560.4075 (C_37_H_54_O_3_N); 423.3618 (C_30_H_47_O); 405.3514 (C_30_H_45_); 295.2419 (C_22_H_31_); 227.1795 (C_17_H_23_); 138.0549 (C_7_H_8_O_2_N); 120.0446 (C_7_H_6_ON)	8.76 × 10^6^
**38**	*O*-aminobenzoyl-bryonolic acid	18.22	C_37_H_53_O_4_N					576.4050	11.5	0.30	439.3584 (C_30_H_47_O_2_); 343.2629 (C_23_H_35_O_2_); 243.1744 (C_17_H_23_O); 138.0551 (C_7_H_8_O_2_N); 120.0445 (C_7_H_6_ON)	2.82 × 10^5^
**39**	Multiflora-trien-diol	18.78	C_30_H_46_O_2_					439.3571	7.5	0.07	407.3310 (C_29_H_43_O); 365.3196 (C_27_H_41_); 323.2375 (C_23_H_31_O); 215.1795 (C_16_H_23_)	3.85 × 10^6^
**40**	Multiflora-dien-diol isomer	19.51	C_30_H_48_O_2_					441.3727	6.5	0.01	409.3465 (C_29_H_45_O); 367.3357 (C_27_H_43_); 311.2373 (C_22_H_31_O); 215.1795 (C_16_H_23_)	3.59 × 10^6^
**41**	Bryononic acid	19.67	C_30_H_46_O_3_					455.3521	7.5	0.17	201.1643 (C_15_H_21_); 173.1327 (C_13_H_17_); 159.1170 (C_12_H_15_); 135.1171 (C_10_H_15_); 121.1015 (C_9_H_13_)	4.98 × 10^5^
**42**	di-*O*-aminobenzoyl-multifloren-triol	20.46	C_44_H_60_O_5_N_2_					697.4576	15.5	−0.76	423.3615 (C_30_H_47_O); 405.3512 (C_30_H_45_); 295.2435 (C_22_H_31_); 203.1794 (C_15_H_23_); 138.0551 (C_7_H_8_O_2_N); 120.0445 (C_7_H_6_ON); 109.1014 (C_8_H_13_)	2.60 × 10^6^
**43**	Multiflora-dien-diol isomer	20.48	C_30_H_48_O_2_					441.3725	6.5	−0.40	409.3464 (C_29_H_45_O); 367.3355 (C_27_H_43_); 215.1796 (C_16_H_23_)	2.94 × 10^6^
**45**	*O*-aminobenzoyl-*O*-acetyl-multifloren-triol	21.16	C_39_H_57_O_5_N					620.4313	11.5	0.63	465.3730 (C_32_H_49_O2); 405.3503 (C_30_H_45_); 203.1797 (C_15_H_23_); 138.0550 (C_7_H_8_O_2_N); 120.0446 (C_7_H_6_ON); 109.1015 (C_8_H_13_)	4.05 × 10^5^
**59**	*O*-aminobenzoyl-*O*-benzoyl-multifloren-triol	24.25	C_44_H_59_O_5_N					682.4452	15.5	0.60	527.3889 (C_37_H_51_O_2_); 423.3621 (C_30_H_47_O); 405.3508 (C_30_H_45_); 295.2435 (C_22_H_31_); 203.1794 (C_15_H_23_); 138.0550 (C_7_H_8_O_2_N); 120.0446 (C_7_H_6_ON); 109.1014 (C_8_H_13_)	8.63 × 10^7^
**60**	*O*-aminobenzoyl-multiflora-dien-diol	25.38	C_37_H_53_O_3_N					560.4101	11.5	−0.48	542.3983 (C_37_H_52_NO_2_); 423.3619 (C_30_H_47_O); 405.3514 (C_30_H_45_); 295.2430 (C_22_H_31_); 227.1795 (C_17_H_23_); 213.1639 (C_16_H_21_); 138.0549 (C_7_H_8_O_2_N); 120.0446 (C_7_H_6_ON)	1.42 × 10^7^
**99**	*O*-aminobenzoyl-*O*-benzoyl-multiflora-dien-diol	31.38	C_44_H_57_O_4_N					664.4355	16.5	−1.55	527.3889 (C_37_H_51_O_2_); 405.3508 (C_30_H_45_); 295.2435 (C_22_H_31_); 138.0550 (C_7_H_8_O_2_N)	1.54 × 10^8^

Note: NL: normalized level.

**Table 5:** Oxylipins and glycolipids were tentatively identified in the EtOH extract of *C. pepo* var. *styriaca* seeds.

Oxylipins												
N°	Compound	R_t_ (min)	Molecular Formula	[M–H]^−^	[(M + HCO_2_H)–H]^−^	RDB	ppm	[M + H]^+^	RDB	ppm	HRMS/MS	MS Peak Intensity (NL)
**1**	Trihydroxy-octadecadienoic acid	2.59	C_18_H_32_O_5_	327.2176		3.5	2.93				309.2066 (C_18_H_29_O_4_); 291.1969 (C_18_H_27_O_3_); 229.1439 (C_12_H_21_O_4_); 211.1332 (C_12_H_19_O_3_); 171.1017 (C_9_H_15_O_3_)	9.85 × 10^5^
**2**	Trihydroxy-octadecenoic acid	3.21	C_18_H_34_O_5_	329.2336		2.5	4.12				311.2227 (C_18_H_31_O_4_); 293.2123 (C_18_H_29_O_3_); 229.1441 (C_12_H_21_O_4_); 211.1333 (C_12_H_19_O_3_); 199.1334 (C_11_H_19_O_3_); 171.1017 (C_9_H_15_O_3_)	8.18 × 10^5^
**25**	Hydroxy-octadecadienoic acid	13.65	C_18_H_32_O_3_	295.2274		3.5	2.09				277.2168 (C_18_H_29_O_2_); 195.1381 (C_12_H_19_O_2_); 171.1016 (C_9_H_15_O_3_)	8.18 × 10^6^
**29**	Hydroxy-octadecenoic acid	14.31	C_18_H_34_O_3_	297.2432		2.5	2.72				279.2325 (C_18_H_31_O_2_); 197.1539 (C_12_H_21_O_2_); 183.1382 (C_11_H_19_O_2_)	2.45 × 10^6^
**31**	Oxo-octadecadienoic acid	14.49	C_18_H_29_O_3_	293.2122		4.5	3.85				275.2013 (C_18_H_27_O_2_); 249.2220 (C_17_H_29_O); 197.1173 (C_11_H_17_O_3_); 185.1173 (C_10_H_17_O_3_)	4.27 × 10^6^
**33**	Oxo-octadecenoic acid	15.56	C_18_H_32_O_3_	295.2281		3.5	4.36				277.2173 (C_18_H_29_O_2_); 251.2380 (C_17_H_31_O); 155.1429 (C_10_H_19_O)	8.15 × 10^5^
**Glycolipids**												
**37**	SQDG (14:0; 16:0)	17.96	C_39_H_74_O_12_S	765.4812		3.5	−1.08				255.2327 (C_16_H_31_O_2_); 225.0075 (C_6_H_9_O_7_S)	2.34 × 10^5^
**75**	DGDG (18:2; 18:2)	27.01	C_51_H_88_O_15_					963.6009 [M + Na]^+^	7.5	−0.68	801.5472 (C_45_H_78_O_10_Na); 683.3612 (C_33_H_56_O_13_Na); 521.3081 (C_27_H_46_O_8_Na)	3.40 × 10^6^
**79**	DGDG (16:0; 18:2)	27.40	C_49_H_88_O_15_					939.6021 [M + Na]^+^	5.5	0.60	777.5496 (C_43_H_78_O_10_Na); 683.3610 (C_33_H_56_O_13_Na); 659.3616 (C_31_H_56_O_13_Na); 521.3104 (C_27_H_46_O_8_Na)	1.04 × 10^6^

Note: abbreviations: SQDGs, sulfoquinovosyldiacylglycerols; DGDGs, digalactosyldiacylglicerols. NL: normalized level.

## Data Availability

The original contributions presented in this study are included in the article/Supplementary Materials. Further inquiries can be directed to the corresponding authors.

## Supplementary Materials

The following supporting information can be downloaded at: https://www.mdpi.com/article/10.3390/foods15122215/s1. Figure S1. Chemical classes of compounds present in the ethanolic extract of the seeds of *C. pepo* var. *styriaca*. Figure S2a–d. Representation of the six PL subclasses. Figure S3a–d. Representation of SL structural types. Figure S4a,b. Representation of the three AG subclasses.
