# Supplementary material for: Cucurbita pepo var. styriaca Seeds: Deep Insights into Polar Lipid Profile
Source: Foods. 2026 Jun 19;15(12):2215. doi: 10.3390/foods15122215 (PMC13298422; doi:10.3390/foods15122215)
Supplement: Supplementary file 1 [file foods-15-02215-s001.zip › foods-4331371-supplementary.pdf]

Supplementary Material

Cucurbita pepo var. *styriaca* seeds: deep insights into polar lipid profile

Annunziata Paolillo[1,2] , Assunta Napolitano[1,*], Francesco Sottile[3], Milena Masullo[1], Sonia Piacente[1,*]

[1] Dipartimento di Farmacia, Università degli Studi di Salerno, via Giovanni Paolo II n. 132, 84084 Fisciano (SA), Italy; anpaolillo@unisa.it ; anapoli@unisa.it; mmasullo@unisa.it; piacente@unisa.it
[2] PhD Program in Drug Discovery and Development, Università degli Studi di Salerno, via Giovanni Paolo II n. 132, 84084 Fisciano, SA, Italy; anpaolillo@unisa.it
[3] Dipartimento di Architettura, Università degli Studi di Palermo, Piazza Marina 61, 90133, Palermo; francesco.sottile@unipa.it

* Correspondence: anapoli@unisa.it (A. Napolitano); piacente@unisa.it (S. Piacente).

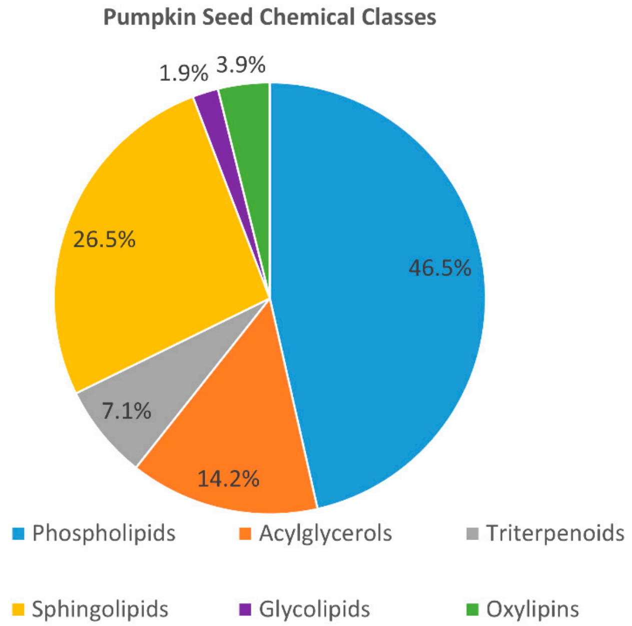

Figure S1. Chemical classes of compounds present in the ethanolic extract of the seeds of *C. pepo* var. styriaca. The pie chart relates the number of components detected for each class in percentages with respect to the total number of compounds detected in Styrian pumpkin seeds.

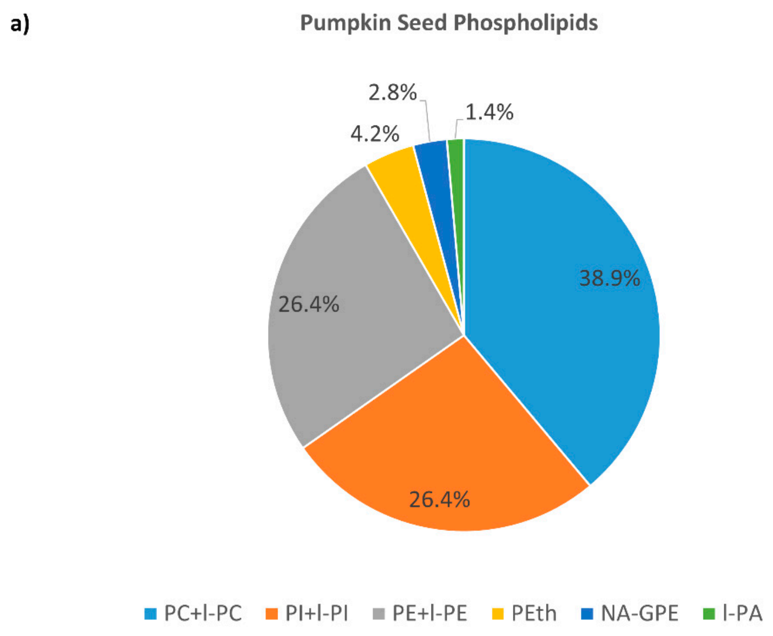

Figure S2a. Representation of the six PL subclasses as a percentage of total number of detected PLs.

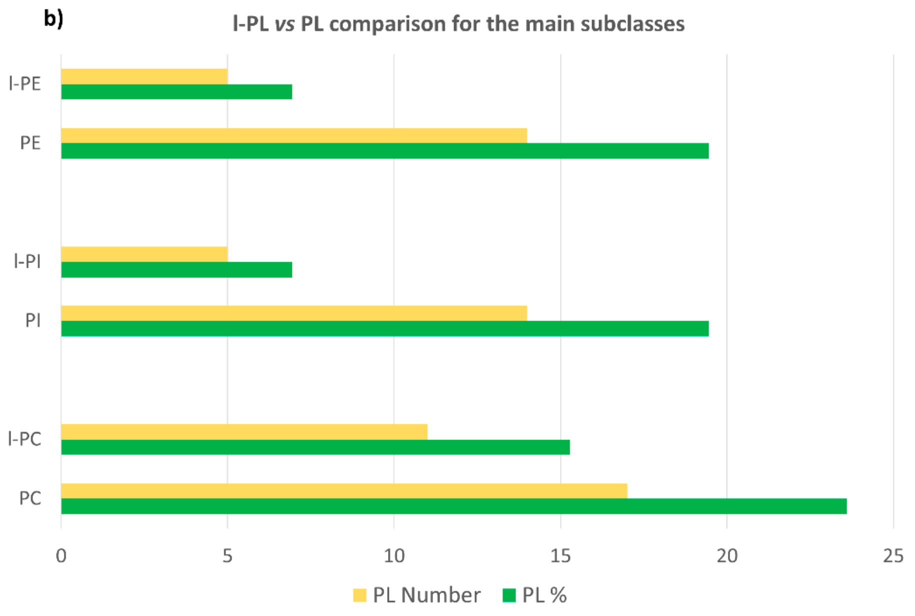

Figure S2b. PL *vs* l-PL comparison for each main subclass: by number of components detected in each subclass and as a percentage of the total number of detected l-PL/PL.

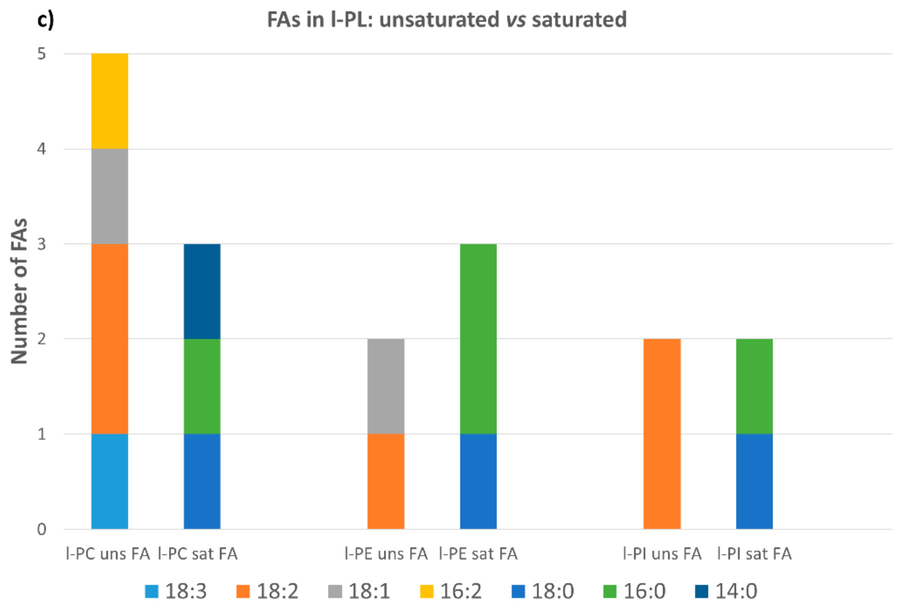

Figure S2c. Number and type of unsaturated (uns) *vs* saturated (sat) FAs in main l-PL subclasses.

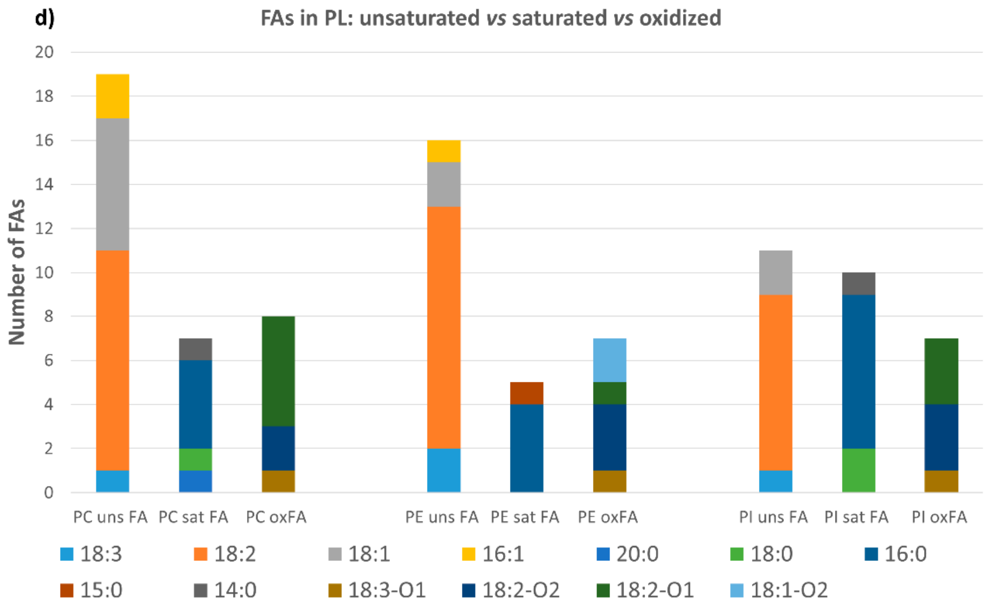

Figure S2d. Number and type of unsaturated (uns) *vs* saturated (sat) *vs* oxidized (ox) FAs in main PL subclasses.

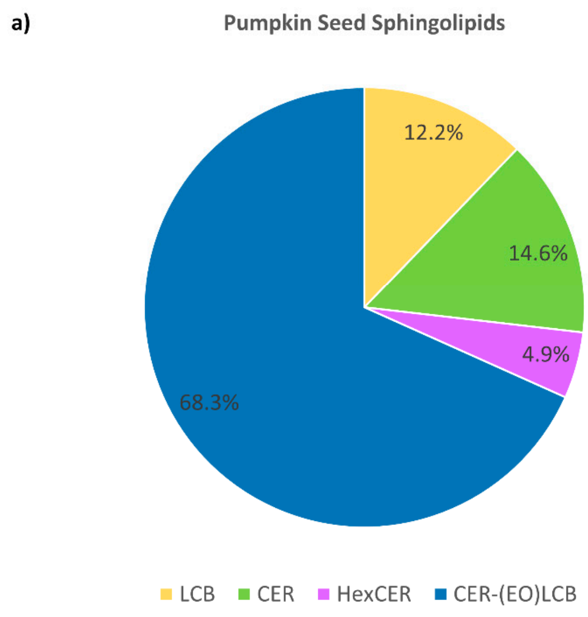

Figure S3a. Representation of SL structural types as a percentage of total number of detected SLs.

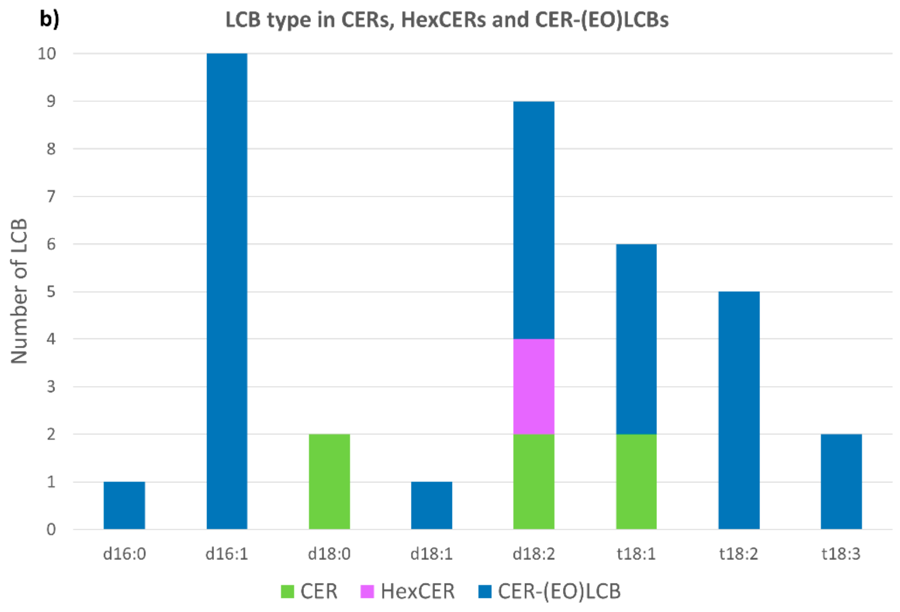

Figure S3b. Number and type of LCBs in the different SL chemical groups.

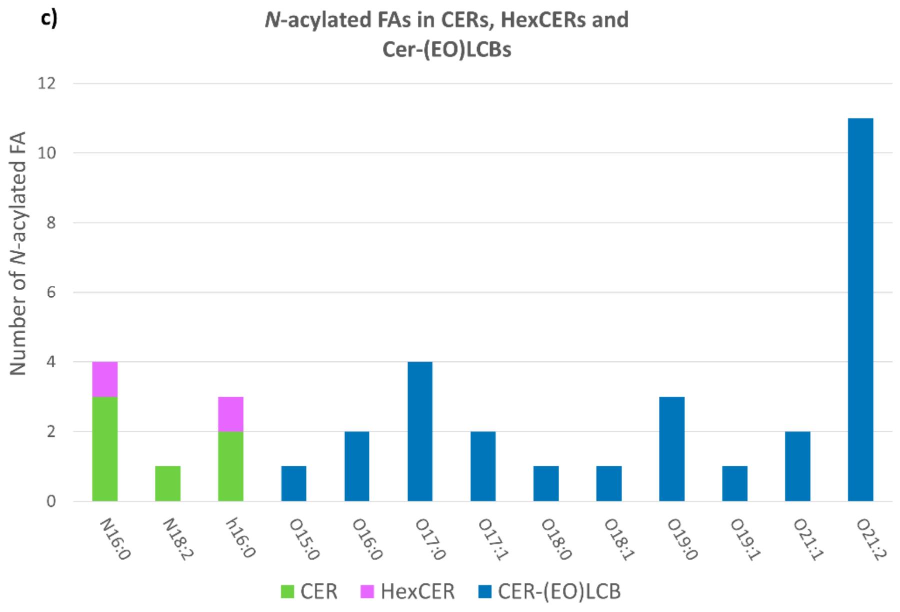

Figure S3c. Number and type of *N*-acylated FAs in the different SL chemical groups with O always being FA h.

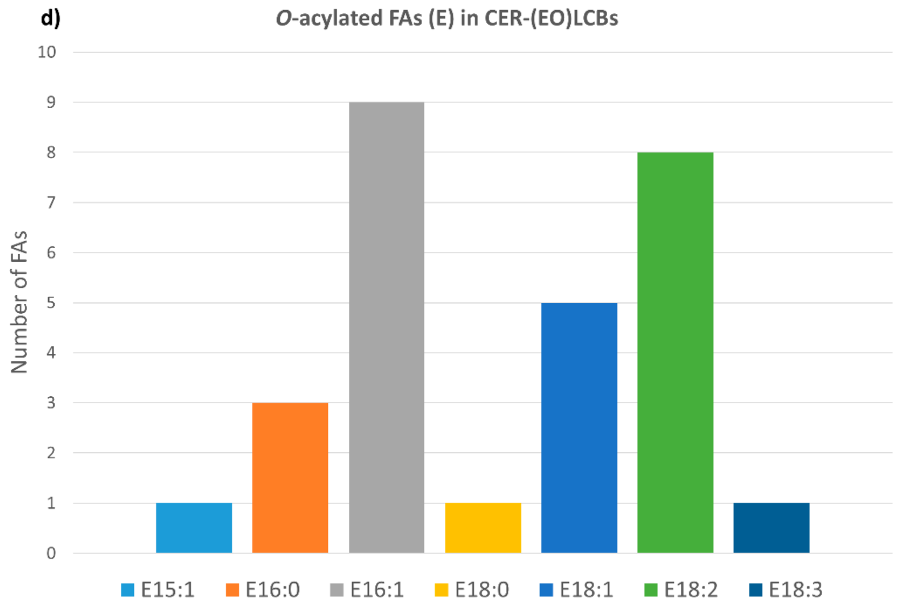

Figure S3d. Number and type of *O*-acylated FAs in Cer-(EO)LCB group.

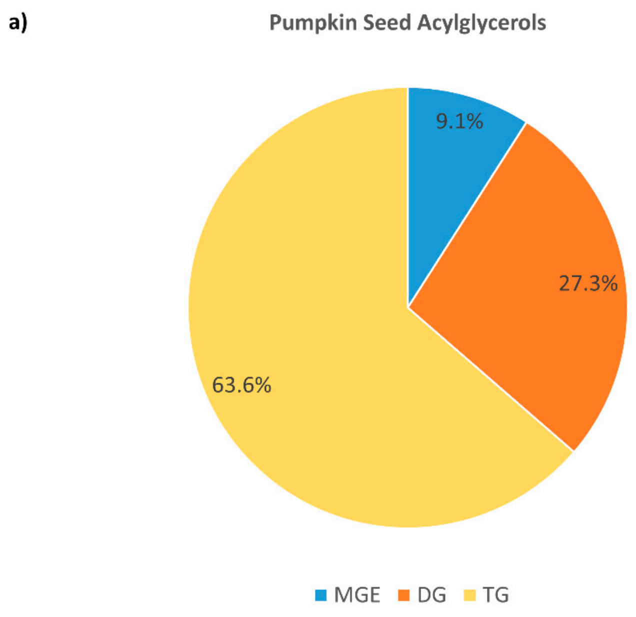

Figure S4a. Representation of the three AG subclasses as a percentage of the total number of detected AGs.

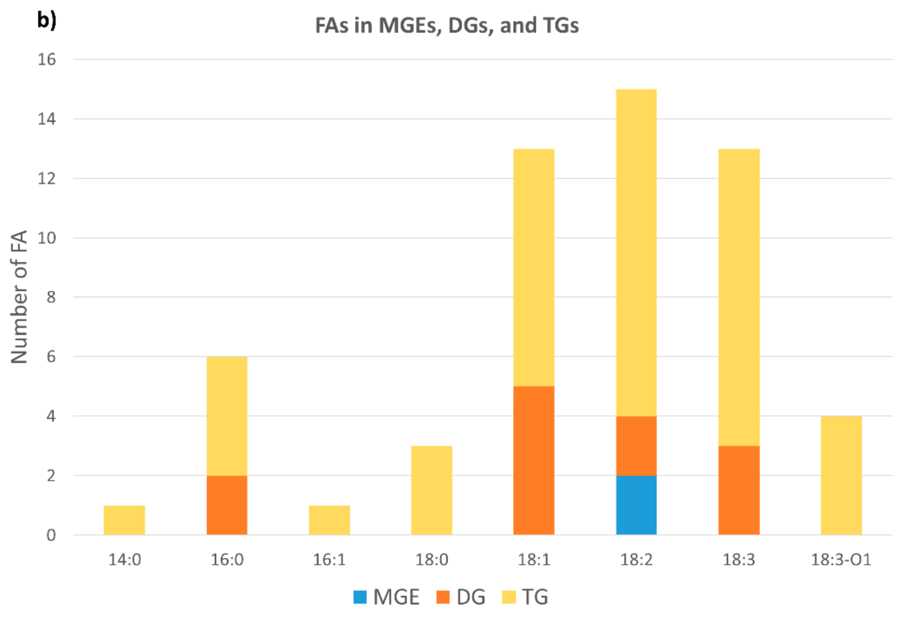

Figure S4b. Number and type of FAs in AG subclasses.
